# Green carbon dots in the era of AI: sustainable synthesis, intelligent drug delivery, advanced diagnostics, and bioimaging

**DOI:** 10.55730/1300-0152.2762

**Published:** 2025-09-09

**Authors:** Emine SEZER, Fulden ULUCAN KARNAK, Sinan AKGÖL

**Affiliations:** 1 Biorege Polymeric Nanosystems & AI Assisted Applications Laboratory, Ege University, İzmir, Turkiye; 2 Department of Computer Engineering, Faculty of Computer and Information Science, Ege University, İzmir, Turkiye; 3 Department of Biochemistry, Faculty of Science, Ege University, İzmir, Turkiye; 4 Sabancı University Nanotechnology Research and Application Center (SUNUM), İstanbul, Turkiye

**Keywords:** Green carbon dots, artificial intelligence, machine learning, deep learning, drug delivery, diagnostics

## Abstract

**Background/aim:**

Green carbon dots (GCDs) are a rapidly developing class of nanomaterials that are revolutionizing various scientific disciplines due to their unique optical properties, low toxicity, and sustainable synthesis. This review offers a comprehensive roadmap for the field, emphasizing the synergy between GCDs and artificial intelligence (AI).

**Materials and methods:**

We begin by detailing the sustainable synthesis of GCDs, highlighting green chemistry principles and the transformative role of AI in optimizing their production. Subsequently, we explore the critical characterization of GCDs, including their structural, optical, and biocompatibility assessment. The core of this study explores the diverse biomedical applications of GCDs, including their integration into intelligent drug delivery systems enhanced by AI, utility in advanced diagnostics and biosensing, and contribution to state-of-the-art bioimaging techniques by deep learning (DL).

**Results:**

Analysis of the literature confirms that AI-driven optimization is crucial for enhancing the scalability and reproducibility of GCD production. Furthermore, the integration of DL models significantly boosts the analytical precision and real-time capabilities of these platforms, validating the profound convergence of the fields.

**Conclusion:**

This review provides a holistic roadmap, concluding that the AI– GCD synergy is indispensable for developing the next generation of smart nanomedicines. Future efforts must prioritize addressing scalability, standardization, and regulatory pathways to accelerate successful clinical translation.

## 1. Introduction

Since their serendipitous discovery in 2004, carbon dots (CDs)—a class of 1–10 nm fluorescent nanomaterials—have gained widespread attention due to their exceptional photoluminescence, aqueous solubility, low cytotoxicity, and facile surface functionalization (Elugoke et al., 2024). These attributes have established CDs as highly adaptable platforms for biomedical and sensing applications. However, concerns over the environmental and health impacts of conventional synthesis methods have driven a paradigm shift toward sustainable alternatives.

In this context, green CDs (GCDs) have emerged as a sustainability-oriented subclass, produced via eco friendly methods from renewable biomass and low-toxicity precursors (Lim et al., 2014; Cui et al., 2021; Lin et al., 2021; Qureshi et al., 2024). GCDs embody the principles of green chemistry through their reduced energy requirements, benign reagents, and alignment with circular material strategies. As such, they represent a crucial evolution in nanomaterial development with meeting the demands of both functional performance and ecological responsibility (Jing et al., 2023).

A crucial semantic clarification is required. The term “ green carbon dots” refers specifically to the sustainability of the synthesis method, while the term “green-emitting CDs” pertains to the photophysical emission wavelength (typically 500–560 nm). Although a single carbon dot may be both green synthesized and green emitting, the 2 descriptors are conceptually and functionally distinct (Jing et al., 2023; Shang et al., 2024). This review focuses on the former green-synthesized GCDs as a material class with transformative potential across biomedical domains.

The convergence of GCDs with artificial intelligence (AI) now opens unprecedented possibilities for precision material design and smart biomedical interfaces. GCDs offer intrinsic biocompatibility and optical versatility, while AI introduces predictive modeling, automated experimentation, and intelligent data interpretation into the nanomaterials pipeline (Etefa and Dejene, 2025). The resulting synergy extends far beyond incremental improvements—it defines a new computational– experimental paradigm where sustainable nanomaterials can be rationally engineered for clinical, diagnostic, and therapeutic use.

This review builds upon this convergence, positioning GCDs not only as functional materials but as intelligent systems within the broader AI-driven landscape. The following sections systematically explore this dual evolution, critically analyzing how AI algorithms—from machine learning (ML) to deep neural networks—enhance the synthesis, characterization, functionalization, and application of GCDs in drug delivery, diagnostics, and bioimaging.

### 1.1. Background and motivation

GCDs, a sustainable subclass of carbon-based nanomaterials, have garnered increasing attention due to their eco friendly synthesis from renewable biomass and their exceptional photophysical and biological properties. Unlike conventional quantum dots that often require toxic metals and hazardous synthetic routes, GCDs can be derived from agricultural waste such as rice husks or citrus peels, natural polymers like chitosan, and plant extracts including tea leaves or turmeric—offering a path toward non toxic, cost-effective, and environmentally responsible nanomaterial production (Lim et al., 2014; Cui et al., 2021; Qureshi et al., 2024). This green synthesis not only aligns with the twelve principles of green chemistry but also supports scalable and biocompatible material development for biomedical use.

From a functional perspective, GCDs offer unique advantages: high photostability, excitation-dependent tunable photoluminescence, rich surface chemistry for modification, and excellent biocompatibility with low cytotoxicity (Dugam et al., 2021; Bhavikatti et al., 2024; Etefa and Dejene, 2025). These features make them ideal for integration into advanced biomedical systems including bioimaging probes, drug nanocarriers, and fluorescent biosensors (Abady et al., 2025; Hasan et al., 2025). However, optimizing GCD performance for specific applications remains experimentally burdensome. The relationship between the synthesis of a material and its properties is governed by nonlinear, multivariate factors such as precursor type, temperature, pH, reaction time, and solvent polarity. Traditional empirical optimization is often resource intensive, time consuming, and generates substantial chemical waste—posing a clear challenge to the sustainability ethos GCDs aim to embody.

Addressing these synthesis challenges requires a paradigm shift. This is where AI technologies can be transformative. AI technologies, particularly ML and deep learning (DL), enable predictive modeling of synthesis outcomes, accelerating discovery while reducing experimental load and environmental impact. Supervised ML approaches can forecast key material properties—such as quantum yield (QY), fluorescence intensity, particle size, surface charge, and cytotoxicity—based on labeled synthesis data, while unsupervised ML can show latent patterns relevant to GCD structure and function (Jin et al., 2023; Roncaglia and Ferrando, 2023; Liu et al., 2024; He et al., 2025). Importantly, CNN-based DL techniques have shown utility in analyzing photoluminescence spectra and microscopy images, enabling high-resolution classification, anomaly detection, and structure–function inference (Döring and Rogach, 2022; Wang et al., 2022). These can be used as a direct feedback metric to guide subsequent synthesis steps in a closed-loop optimization framework.

Beyond predictive capacity, AI contributes directly to sustainability. By reducing the number of necessary experiments through in silico screening, active learning, and reinforcement-based design, AI-driven synthesis minimizes material consumption, energy use, and hazardous waste generation. Quantitatively, such methods have the potential to reduce experimental iterations by over 80%, leading to proportional decreases in solvent waste, energy demand, and experimental effort—making green nanomaterial research feasible and scalable (Alagumalai et al., 2022; Betz et al., 2023; Xie et al., 2025).

Despite the potential of AI, its impact on GCD research is hampered by data infrastructure limitations, a lack of methodological standardization, and interpretability challenges, especially within complex biomedical applications. AI model efficacy hinges on access to large, high-quality, and standardized datasets, which are currently scarce in GCD research. The heterogeneity of synthesis protocols, varied experimental reporting, and absence of public benchmarking datasets restrict the generalizability and reproducibility of predictive models. Furthermore, while tools like SHapley Additive ExPlanations (SHAP) and local interpretable model-agnostic explanations (LIME) offer insights into feature importance, they often struggle to capture the intricate, nonlinear dependencies of deep neural networks, particularly crucial in safety-critical areas such as drug delivery and diagnostic biosensing (Oviedo et al., 2022; Jin et al., 2023). Overcoming these limitations will require collaborative efforts in data curation, community-wide adoption of standardized ontologies and reporting formats, and the development of explainable AI (XAI) frameworks specifically tailored for the nanomaterials and bioengineering domains (Mishra et al., 2023a).

**Figure 1:**
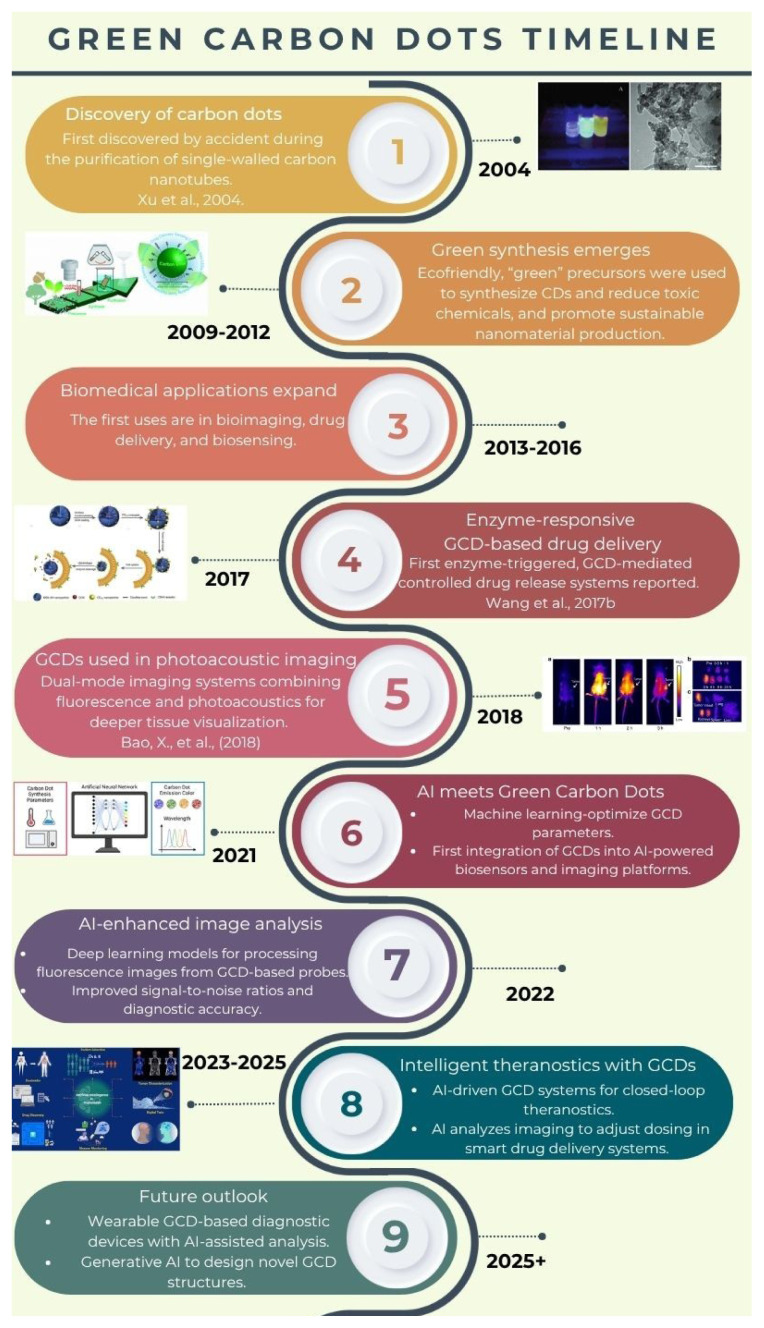
Historical timeline of GCDs: key milestones and developments.

Taken together, the convergence of GCDs and AI offers a powerful platform for next-generation biomedical systems: materials that are not only green and biocompatible but also intelligently designed, optimized, and validated through data-driven strategies. The evolution of GCD technology, from its initial discovery to the current integration with AI frameworks, is chronologically summarized in Figure 1, highlighting major milestones and paradigm shifts over the past 15 years.

### 1.2. Scope and objectives

Driven by the limitations of traditional methods and the urgent need for sustainable solutions, the integration of GCDs with AI offers a paradigm shift in materials science (Kurian and Paul, 2021; Dhumal et al., 2024). Integrating AI into GCD synthesis, characterization, and biomedical applications is crucial, driven by the need for sustainable nanomaterials and the inherent complexities of GCD optimization. AI, through ML and DL, will revolutionize GCD development by optimizing synthesis parameters, predicting material properties, and automating processes, thereby minimizing resource consumption and accelerating eco friendly discovery. Furthermore, AI will enable intelligent drug delivery with real-time monitoring and personalized strategies, and enhance advanced diagnostics by interpreting complex biosensor and bioimaging data. Holistic AI integration across the GCD lifecycle will manage complexity, accelerate innovation, and ensure nanotechnology contributes to a more sustainable and healthier future (Wasilewski et al., 2024; Chakro borty et al., 2025)

This review aims to systematically explore how AI techniques are transforming the design, optimization, and application of GCDs across biomedical domains. While the promise of AI-assisted nanomaterial development and the rationale for focusing on GCDs is mentioned above, the subsequent sections explore specific areas where this convergence has shown the most traction and future potential.

To that end, this review centers on 4 interconnected and progressive domains: sustainable synthesis, intelligent drug delivery, advanced diagnostics, and bioimaging. Collectively, these represent the full functional lifecycle of GCD-based systems in biomedicine (Jin et al., 2023;; Qureshi et al., 2024). These domains are not only aligned with pressing scientific and clinical needs but also reflect areas where AI-driven methods have made substantial contributions in terms of predictive modeling, experimental efficiency, and translational viability (Zeng et al., 2023; Liu et al., 2024; Patyal et al., 2025).

Accordingly, the review is structured around 4 core objectives: 1) to analyze sustainable GCD synthesis methods, with emphasis on how AI models are used to predict and optimize key physicochemical parameters such as fluorescence intensity, QY, particle size, and biocompatibility; 2) to explore AI-enabled strategies in drug delivery, including in silico interaction modeling, controlled release kinetics, and personalized therapeutic design; 3) to assess diagnostic and imaging applications, focusing on how AI improves sensitivity, multiplexing, and data interpretability in biosensing and bioimaging platforms; and 4) to identify existing limitations and translational barriers, particularly regarding data availability, model generalizability, and ethical or regulatory issues in the deployment of AI-driven GCD systems (Oviedo et al., 2022; Jin et al., 2023).

From this point onward, the review is organized into the following sections: an exploration of sustainable synthesis routes and AI-assisted optimization (section 2), in-depth discussions on material characterization and toxicity assessment (section 3), therapeutic design (section 4), diagnostic platforms (section 5), and bioimaging strategies (section 6). Section 7 focuses on AI-driven automation and data modeling , and the review concludes with a critical examination of regulatory hurdles, emerging technologies, and future perspectives ( sections 8 and 9).

## 2. Sustainable synthesis of green carbon dots

The pursuit of sustainable nanomaterial synthesis has become a central concern in modern materials science, particularly in biomedical applications where safety, scalability, and ecological impact are tightly intertwined (Prasanna Babu Racheeti, 2024). GCDs have emerged as a promising class of fluorescent nanomaterials that align with green chemistry principles by utilizing eco friendly precursors, mild reaction conditions, and minimal use of toxic reagents (; Etefa and Dejene, 2025). Their synthesis methods often draw from renewable feedstocks, including agricultural waste, food by products, and plant-based materials, offering a low-cost and biodegradable alternative to conventional CDs . This section provides an overview of GCD synthesis strategies, beginning with their chemical and environmental foundations, and progressing toward AI-assisted optimization workflows that reduce experimental burden and improve reproducibility. Rather than presenting an exhaustive inventory of synthesis protocols, the focus is placed on the green chemistry rationale, synthetic versatility, and the opportunities for ML-driven design space exploration.

### 2.1. Green chemistry principles

GCDs align with the twelve principles of green chemistry, primarily through their reliance on renewable resources, waste prevention, and energy efficiency (Anastas and Eghbali, 2009; Hasan et al., 2025). Unlike conventional quantum dots that use heavy metals and hazardous solvents, GCDs are synthesized from sustainable biomass like rice husks, citrus peels, and other organic waste. These bio sources provide inherent functional groups (e.g., amino, carboxyl, and hydroxyl) and natural photoluminescent properties due to nitrogen, sulfur, and oxygen heteroatoms , making them ideal for the large-scale, economical, and non toxic production of GCDs. Furthermore, GCD synthesis uses mild thermal techniques such as hydrothermal or microwave-assisted production, minimizing energy consumption and carbon footprint, while the use of green solvents like water and ethanol enhances environmental and toxicological safety (Dong et al., 2024; Ahmad et al., 2025; Aksu and Güzdemir, 2025).

Crucially, the photophysical characteristics of GCDs, including their QY, emission wavelength, and surface charge, are highly tunable but complex, varying significantly with precursor composition, reaction pH, temperature, and time. This makes GCD synthesis an ideal candidate for AI-assisted modeling, where ML algorithms can identify nonlinear relationships between synthesis parameters and resulting material properties, thus reducing waste and experimental iterations (Jin et al., 2023; Zeng et al., 2023).

### 2.2. Synthesis approaches

The evolving field of GCDs emphasizes sustainable and scalable synthesis from renewable biomass and natural organic materials, adhering to green chemistry principles to minimize environmental impact and toxicity. While traditional top-down methods (e.g., arc discharge and laser ablation) exist, their high costs, hazardous ingredients, and purification challenges limit their green application. Bottom-up methodologies are the primary focus for GCD synthesis. This approach offers superior control over GCD properties and are more advantageous for sustainable mass production due to better yields, scalability, and cost effectiveness. Hydrothermal/solvothermal methods are favored for their simplicity and adaptability with diverse biomass. Pyrolysis is an eco friendly process, converting biomass into luminous GCDs. While microwave-assisted synthesis is fast, ultrasonic approaches are cheaper and greener, though both face precision and efficiency challenges. Templates aid size control and reduce aggregation but pose removal difficulties. Current GCD synthesis research focuses on refining reaction parameters, finding new natural sources, and co doping for enhanced properties, with the aim of developing efficient, low-cost, scalable materials. Top-down and bottom-up methods are compared Table, considering parameters such as yield, scalability, cost, purification demand, and sustainability. All references for this information and more details are cited in row 7 of Table.

**Figure 2:**
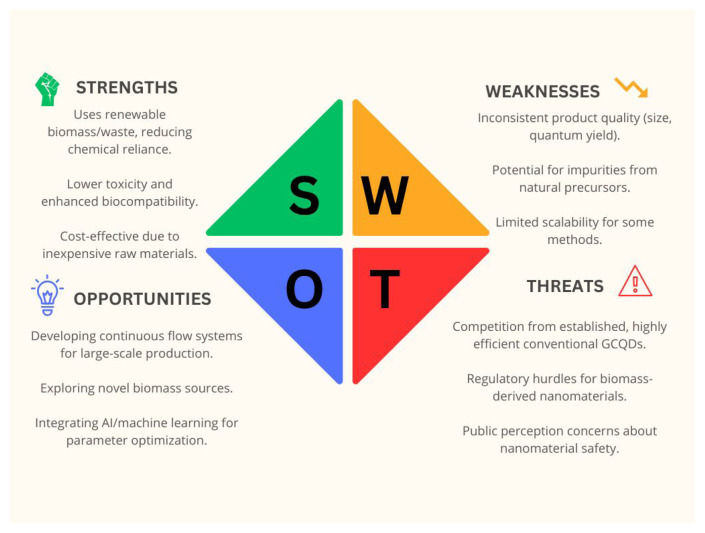
SWOT analysis diagram of GCD synthesis methods.

To comprehensively evaluate the viability and potential of current GCD synthesis methodologies, a strengths, weaknesses, opportunities, and threats (SWOT) analysis is presented in Figure 2, offering a structured perspective on their advantages, limitations, and future outlook .

Despite the variety of synthetic strategies available, achieving batch-to-batch reproducibility, precise control over photoluminescent features, and scalability for clinical-grade applications remains challenging. These limitations highlight the need for data-driven approaches that can map the multidimensional synthesis space and optimize protocols accordingly. This will be explored in detail in the following section.

### 2.3. AI- assisted synthesis optimization

The synthesis of GCDs is a multidimensional process influenced by several interdependent parameters, including precursor type, reaction time, temperature, pH, dopant concentration, and solvent conditions. Traditional trial-and-error methods remain inefficient for identifying optimal synthesis conditions, especially when the goal is to tailor GCD properties for downstream biomedical applications (Zhang et al., 2022). Furthermore, the vast chemical design space and high cost of experimentation hinder rapid material discovery.

To address these challenges, ML and AI techniques have been increasingly used to streamline GCD synthesis through data-driven optimization. Supervised regression models, such as support vector regression (SVR), decision trees, and ensemble algorithms, have been used to predict fluorescence intensity, QY, particle size, and surface charge based on synthesis parameters (Senanayake et al., 2022; Liu et al., 2024; Chakro borty et al., 2025). These models not only accelerate parameter tuning but also identify complex nonlinear relationships that are often overlooked in empirical studies.

**Figure 3:**
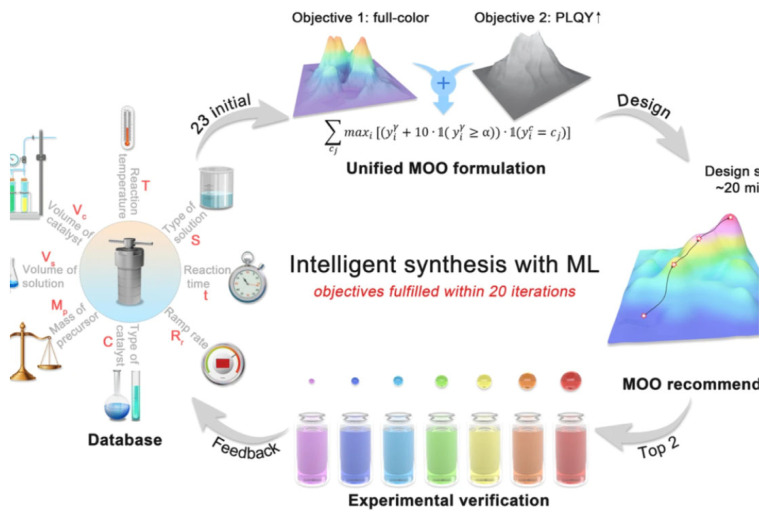
Schematic of a closed-loop AI-assisted synthesis workflow (adapted from Guo et al., 2024). This schematic illustrates a multi objective optimization (MOO) approach driven by machine learning to efficiently achieve desired properties like full-color emission and high photoluminescence quantum yield (PLQY). The process involves an iterative cycle of initial data, AI-guided design, experimental verification, and data feedback to continuously refine synthesis conditions.

Building upon this, DL methods like convolutional neural networks (CNNs) and recurrent neural networks (RNNs) can handle large, high-dimensional data from time-resolved spectroscopy, reaction imaging, and multi modal process monitoring. When integrated into closed-loop experimental platforms, these AI systems can autonomously suggest and execute new experimental conditions based on real-time analysis. This paradigm is illustrated in Figure 3, which depicts an intelligent materials discovery cycle combining robotic synthesis, in situ characterization, and DL inference (Guo et al., 2024; Zhang et al., 2025).

Such closed-loop frameworks exemplify a shift from passive prediction to autonomous exploration, wherein GCD synthesis becomes a dynamic, AI-guided process. The integration of XAI methods, such as SHAP and LIME, further enhances interpretability by showing the relative importance of input variables, enabling scientists to validate and trust model outputs in safety-critical biomedical contexts (Yang et al., 2023).

Importantly, these AI-driven approaches align with the principles of green chemistry by reducing solvent usage, minimizing waste, and lowering energy consumption. For instance, iterative modeling has been shown to reduce the number of experimental runs by up to 80%, thereby accelerating discovery while simultaneously decreasing environmental footprint (Lim et al., 2020).

In summary, AI-assisted optimization is not merely a computational shortcut—it represents a transformative paradigm in sustainable nanomaterial development. It enables rational GCD design by combining predictive modeling with automated experimentation, laying the foundation for scalable, cost-effective, and application-specific synthesis pathways.

### 2.4. Surface functionalization of GCDs

Surface functionalization of nanomaterials, like GCDs, is incredibly important for biomedical applications (Farshbaf et al., 2018). This process introduces groups such as –COOH, –NH_2_, and –OH, drastically improving aqueous solubility and biocompatibility. These functional groups also act as versatile anchors, allowing the attachment of specific molecules. This means we can precisely design nanomaterials for tasks like targeted drug delivery, attaching diagnostic agents for enhanced imaging, or developing sensitive biosensors. Ultimately, this precision leads to better efficacy and fewer side effects across a wide range of medical technologies (Kaymaz et al., 2023; Kar et al., 2024). Doping strategies further refine GCD properties by incorporating heteroatoms (e.g., N, S, and P) or metal ions. Heteroatom doping introduces defect states that modulate the electronic structure, enhancing QY by facilitating radiative recombination pathways and suppressing non radiative decay. Concurrently, metal ion doping can enhance optical responsiveness or bestow catalytic properties relevant for imaging or therapeutic applications (Ghosh et al., 2020; Nguyen et al., 2022; Kong et al., 2024; Yu et al., 2025). In particular, doping with hydrophilic functional groups (e.g., –NH_2_ and –SO_3_ H) significantly boosts dispersibility and colloidal stability in physiological environments (Ala et al., 2025; Esmaeili et al., 2025; Yu et al., 2025), which are critical for reducing aggregation, lowering immunogenicity, and prolonging in vivo circulation time (Kyriakides et al., 2021).

Traditional approaches to surface engineering are labor-intensive and lack predictability due to the inherent complexity of GCD interfaces. Here, AI—especially ML—plays a transformative role. Supervised algorithms such as random forest and gradient boosting can be trained on physicochemical and biological datasets to predict outcomes like ligand binding affinity, cellular uptake efficiency, and bio distribution profiles (Gao et al., 2024a; Pandey et al., 2024). In their 2025 review, Ou et al. highlight how computational tools, particularly generative AI, are being utilized for the *de novo* design of novel peptide binders with therapeutic potential. This AI-driven approach represents a significant advancement over traditional methods of discovering or utilizing existing targeting ligands such as folic acid and aptamers. Similarly, Park et al. (2023) reported a closed-loop ML platform that dynamically fine-tuned ligand presentation based on feedback from binding assays, significantly improving the selectivity and therapeutic index.

## 3. Characterization and properties

Comprehensive characterization of GCDs is essential to link their structural and optical features with their performance in biomedical applications. Morphological traits such as particle size and dispersion are typically analyzed using transmission electron microscopy (TEM) and scanning electron microscopy (SEM), while X-ray photoelectron spectroscopy (XPS) and Fourier transform-infrared spectroscopy (FTIR) provide insights into surface chemistry and functional groups critical for bioconjugation. Raman spectroscopy elucidates defect structures and graphitic content, whereas ultraviolet–visible (UV–Vis) and photoluminescence spectroscopy are used to assess absorption and emission profiles. Due to the high sensitivity of GCD properties to synthesis parameters, advanced data analysis methods including AI-assisted spectral interpretation and image-based feature extraction are gaining popularity in this field.

### 3.1. Structural and optical characterization

Characterizing GCDs is essential, as their physicochemical properties directly dictate performance, offering key insights for controlled synthesis and optimal functionality. AI significantly improves materials science by boosting predictive modeling of material properties and streamlining data analysis in techniques like X- ray diffraction, Raman spectroscopy, scanning probe microscopy, and electron microscopy, ultimately enhancing accuracy and pattern identification from large datasets (Chávez-Angel et al., 2025). The most fundamental methods used for this are detailed below. TEM and SEM are crucial for characterizing the morphology and size distribution of GCDs. TEM offers high-resolution insights into individual GCDs, showing their quasi spherical shape, size, and internal crystalline structure, enabling the study of polydispersity. SEM provides complementary information on GCD distribution and aggregation behavior on substrates, capturing details on shape, topography, and chemical composition, although with lower resolution than TEM and limitations in large-scale imaging ( Jiang et al., 2019; Mansuriya and Altintas, 2021; Díaz-García et al., 2024).

**Figure 4:**
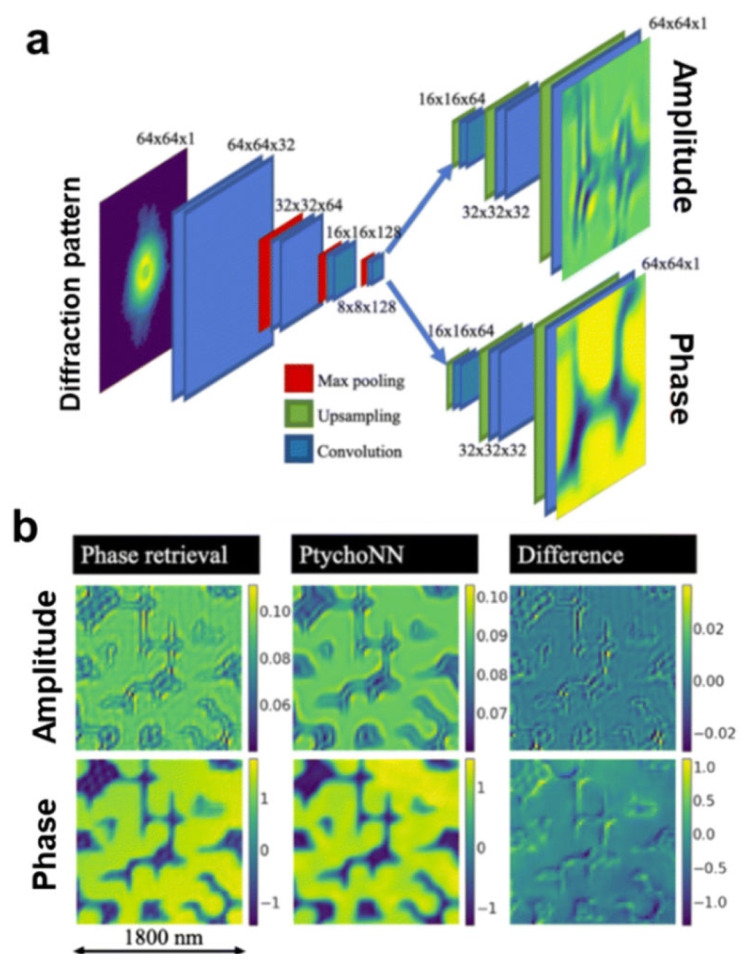
Illustration of ML is transforming high-dimensionality data analysis in electron microscopy. ( a) PtychoNN, an encoder-double decoder CNN, specifically designed for the ptychography reconstruction of amplitude and phase from single diffraction patterns. (b) PtychoNN achieves higher fidelity in wave information reconstruction while operating up to 300 times faster than traditional iterative phase retrieval algorithms, enabling capabilities like real-time ptychography for challenging samples.

Manual image analysis of TEM and SEM images can be being time consuming, labor intensive, and susceptible to human error. This severely impedes the rapid, reproducible characterization necessary for high-throughput synthesis and quality control. To overcome these limitations, computer vision models, particularly CNNs , are increasingly being adopted to automate GCD image analysis. Despite the promise of transfer learning with CNNs in microscopy for tasks like classification and object detection, its broader application is currently restricted. Moreover, CNNs often lack generalizability, meaning models trained for a specific supervised task cannot easily be adapted to diverse image analysis problems (Lu et al., 2022). Consequently, while ML has notably advanced various electron microscopy techniques, its full integration within the electron microscopy community for GCD-specific applications still faces considerable challenges. Botifoll et al. (2022) highlighted PtychoNN, an encoder– double decoder CNN, for its ability to rapidly reconstruct high-fidelity amplitude and phase from single ptychography diffraction patterns, achieving up to 300 times faster results than traditional methods. This breakthrough enables real-time ptychography, proving highly beneficial for dose-sensitive and thick samples (Figure 4).

These AI pipelines significantly accelerate microscopy analysis, enabling rapid, precise quantification of features like particle size, agglomerations, and lattice defects, drastically reducing analysis time. However, their performance relies heavily on high-quality, abundant training data, making them vulnerable to misinterpreting imaging artifacts. Therefore, rigorous model validation and standardized, open-access image datasets are crucial for reliable deployment.

XPS is a powerful surface-sensitive technique vital for characterizing the elemental composition and chemical states of GCD surfaces, identifying elemental compositions. This information is critical for understanding surface functionalization and carbon atom hybridization. While advancements allow XPS analysis under near-ambient pressures and with micron-scale resolution, its inherent surface sensitivity makes it highly vulnerable to contamination from adsorbed species like water and hydrocarbons (Sarma et al., 2013; Arble et al., 2018; Lou et al., 2021; Bellucci and Speranza, 2022; Speranza, 2022; Saputra et al., 2024). The integration of AI, especially LLM-informed models, has shown promise in interpreting complex spectral data by identifying subtle bonding shifts and constructing predictive models that correlate elemental ratios with synthetic parameters (Chávez-Angel et al., 2025). This automated interpretation facilitates faster and more reproducible surface analysis while reducing the manual annotation workload.

FTIR is a valuable technique for identifying the functional groups present on the surface of GCDs. By analyzing the absorption of infrared light by molecular bonds, FTIR shows specific stretching and bending vibrations corresponding to groups like hydroxyl (– OH), carboxyl (– COOH), amine (– NH2), and carbonyl (C=O). These functional groups, often including ether or epoxy, are critical for determining the solubility, stability, and reactivity of GCDs . For instance, characteristic peaks at 1724 cm^−1^ and 3307 cm^−1^ confirm carboxyl groups, while 1579 cm^−1^ and 1097 cm^−1^ indicate ether linkages and double bonds, respectively. FTIR is favored for its affordability, ease of use, and speed. However, it provides limited detailed structural information and cannot directly detect metal heteroatom doping (Singh et al., 2018; Shabbir et al., 2023; Mu et al., 2025). AI-driven clustering and pattern recognition algorithms can automate the spectral deconvolution of FTIR data, enabling rapid annotation of chemical motifs and identification of outliers, especially in high-throughput settings.

Raman Spectroscopy is a powerful, non destructive technique for characterizing the carbon lattice structure, defects, and crystalline or amorphous nature of GCDs. It offers distinct advantages, including no sample preparation and micron-scale spatial resolution, making it promising for crystallite size estimation. Key to Raman analysis of carbonaceous materials are the D band (ID) (around 1352 cm^−1^ ), indicating disordered carbon, and the G band (IG) (around 1585 cm^−1^ ), associated with graphitic carbon. The ID/IG ratio quantifies the degree of defects and disorder within the carbon structure. However, accurately fitting Raman spectra for highly disordered carbons (La ≤ 2 nm) remains a significant challenge due to the lack of a universally accepted methods and potential for subjective results (Merlen et al., 2017; Puech et al., 2019; Kanwal et al., 2022). Integrating AI, specifically ML algorithms, could revolutionize Raman data analysis by developing more robust and standardized fitting protocols for disordered carbons, automatically identifying subtle spectral changes and correlating them with GCD properties, ultimately accelerating materials characterization and design. Standardized chemometric pipelines—from experimental design and rigorous baseline correction to dimensionality reduction, cross-validation, and model transfer—have been proposed by Guo et al. (2021) and provide a robust template for integrating machine-learning algorithms into Raman analysis of carbon-rich nanomaterials, including GCDs.

**Figure 5:**
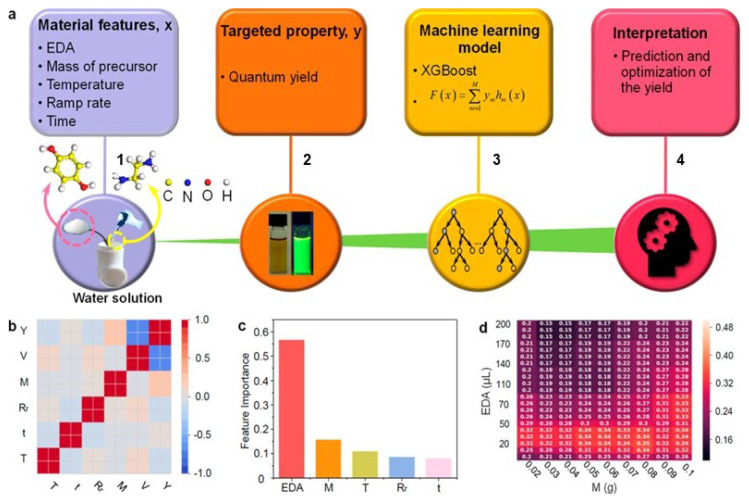
ML -guided optimization of CD synthesis using Gaussian process regression. The workflow illustrates the iterative prediction and refinement of synthesis parameters (e.g., ethylenediamine (EDA), precursor ratios, temperature, and time) to maximize quantum yield (QY) and control particle size. Adapted from Han et al. (2020).

UV–Vis absorption spectroscopy plays a central role in assessing the optical properties of GCDs, quantifying their absorption of ultraviolet and visible light and evaluating the optical transitions of GCDs. Characteristic absorbance features in the 250–300 nm range correspond to π–π* transitions of C=C bonds, while n–π* transitions of C=O groups appear between 270–360 nm (Das et al., 2021; Kavgacı et al., 2023; Yang et al., 2023). The hallmark property of GCDs—excitation-dependent photoluminescence (PL)—stems from quantum confinement, surface defects, and heteroatom doping. Red-emitting GCDs are particularly advantageous for in vivo bioimaging due to deeper tissue penetration and minimal photodamage (Ding et al., 2016; Wang et al., 2017a; Jorns et al., 2021). AI-based regression models have been used to map PL characteristics and QY against synthetic parameters and functional group composition, guiding the design of tailored emissive nanomaterials (Nie et al., 2014, Anpalagan et al., 2024; Xing et al., 2024). As summarized in Figure 5, Han et al. (2020) showed how ML-guided synthesis can predict and optimize both QY and particle size in carbon dot systems produced fromethylenediamine (EDA) using Gaussian process regression (GPR) models to iteratively refine synthesis conditions based on experimental feedback.

The integration of AI into GCD characterization offers a powerful solution, enabling high-throughput, accurate, and interpretable analysis of their features. This not only streamlines materials development and enhances reproducibility but also provides precise control over GCD luminosity. By predicting and correlating excitation –emission profiles and QY with synthesis parameters like precursor, solvent, temperature, and reaction time, AI accelerates the rational design of highly emissive, tailored, luminescent nanomaterials for advanced bioimaging, sensing, and diverse applications (Mansuriya and Altintas, 2021; Wang and Lu, 2022; Ateia et al., 2024; Kar et al., 2024; Mohanaraman and Chidambaram, 2024; Xu et al., 2024).

### 3.2. Biocompatibility and toxicity assessment

Evaluating the toxicity and biocompatibility of GCDs is crucial for their safe biomedical application, necessitating rigorous in vitro and in vivo testing. In vitro cytotoxicity tests (e.g., MTT and CCK-8) provide initial assessments of dose-dependent effects and IC50 on various cell lines (e.g., HeLa, fibroblasts, and immune cells). Further in vitro analyses include genotoxicity, oxidative stress indicators, and inflammatory response assessments (Dias et al., 2019; Skrzydlewski et al., 2022; Matotoka et al., 2025; Tolosa et al., 2015). However, in vitro models cannot fully replicate biological complexity, necessitating in vivo models for comprehensive nanotoxicity evaluation. These are crucial for assessing factors like administration route, biodistribution, biodegradability, and the induction of developmental abnormalities, which in vitro settings cannot adequately address. Therefore, reliable in vivo models are essential to bridge the gap between cell models and small mammalian studies in nanomaterial toxicity assessment (Kang et al., 2013; Dias et al., 2019). Due to its quick development, genetic resemblance to humans, adaptability for real-time observation, and economical rearing, the zebrafish (*Danio rerio*) is a widely used model for the in vivo toxicity assessment of novel nanomaterials, including GCDs. The zebrafish embryo toxicity (ZET) experiment is a reliable technique for determining developmental defects and environmental toxicities (Roper and Tanguay, 2018; Teame et al., 2019). Its advantages in swiftly assessing developmental defects and acute toxicity complement comprehensive rodent studies that are crucial for evaluating systemic responses, biodistribution, and long-term toxicity in major organs (liver, kidney, spleen, and brain) (Garcia et al., 2016; Chahardehi et al., 2020; Kumar et al., 2023; Tegafaw et al., 2025).

Careful surface functionalization is crucial for GCDs to minimize toxicity and maximize biological interactions. This process enhances colloidal stability and water solubility, preventing aggregation, reducing non specific protein adsorption, and extending circulation half-life. Such modifications also mitigate oxidative stress and inflammatory responses, making GCDs safer for biomedical applications (Guerrini et al., 2018; Xuan et al., 2023). Biocompatible polymers like polyethylene glycol (PEG) can be covalently bonded to GCDs to improve biodistribution, cellular uptake, and clearance, while reducing immunological reactions and non specific protein absorption (Suk et al., 2015; Havrdova et al., 2016). While PEG mainly boosts photoluminescence QY (PLQY) and stability, it can also influence emission wavelength. In contrast, cationic polyethylene imine (PEI) is often used to enhance membrane permeability and provide positive surface charges for applications like gene transfer, despite potential biological interactions. Ultimately, surface functionalization profoundly customizes the chemical reactivity of GCDs and broadens their applications by attaching distinct chemical groups, frequently altering their emission wavelengths (e.g., from green to blue) and expanding their utility (Sakdaronnarong et al., 2020).

AI can significantly accelerate and enhance the analysis of high-dimensional toxicity data, including imaging data from developmental defect screenings and complex omics data from systemic responses. ML and DL models can predict cytotoxicity based on physicochemical descriptors like particle size, surface charge, and functionalization profiles (Ji et al., 2022; Shirokii et al., 2023). Modern regression models provide nuanced, concentration-dependent cytotoxicity predictions, reducing reliance on animal studies. For example, Shirokii et al. (2023) developed an ML pipeline achieving high predictive performance (Q ^2^ = 0.86, RMSE = 12.2%) for in vitro cytotoxicity, accelerating material design. Furthermore, ensemble models like XGBoost and random forest integrate diverse data to predict toxicity in real time (Yan et al., 2023), and vision transformers (ViT) correlate fluorescence intensity with oxidative stress biomarkers for superior accuracy in neuroimaging^1^ . These AI-driven strategies are increasingly complemented by mechanistic modeling and ab initio simulations, providing a multiscale understanding of degradation and reactive oxygen species (ROS) generation (Wang et al., 2021; Cave et al., 2025). By integrating AI into toxicity evaluation, the field can move toward predictive, interpretable, and scalable toxicology, supporting the rational design of safer GCDs for biomedical applications (Martin et al., 2023).

**Figure 6:**
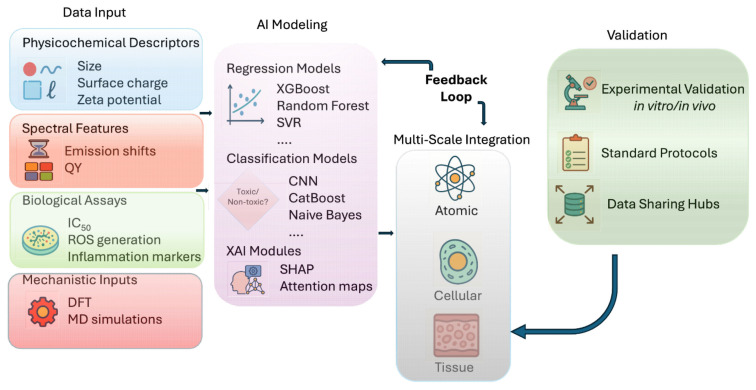
Integrated pipeline for AI-enabled nanotoxicity assessment of GCDs. This figure shows a complete workflow for predicting and validating the toxicity of GCDs. The pipeline combines a wide range of data inputs with various AI models and uses a feedback loop for continuous improvement. The final predictions are validated at atomic, cellular, and tissue scales through standard experimental protocols.

The conceptual framework of AI-driven nanotoxicity assessment for GCDs is illustrated in Figure 6, integrating diverse input modalities (physicochemical, spectral, biological, and mechanistic) into regression and classification pipelines. These models inform multiscale toxicity predictions—ranging from atomic-level interactions to tissue-level responses—and are iteratively refined through experimental validation and data-sharing mechanisms to enhance model generalizability and trustworthiness.

**Figure 7:**
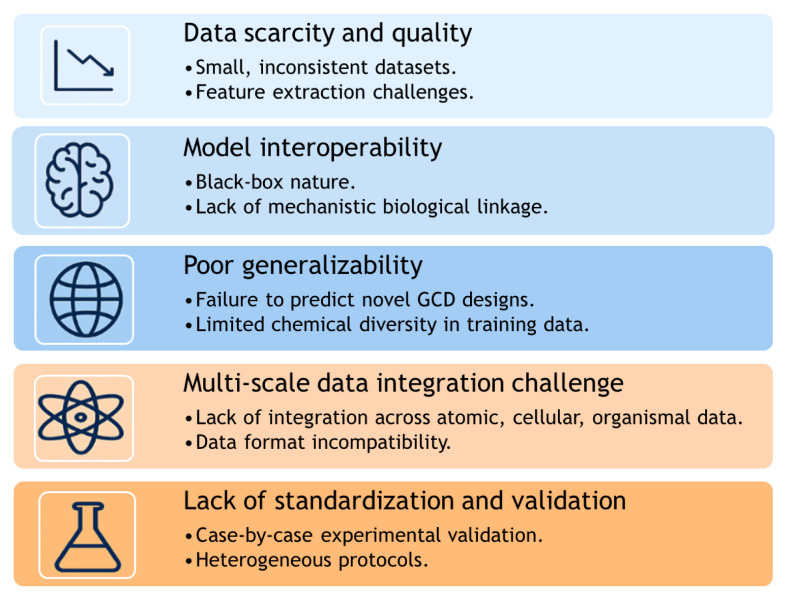
Major challenges and limitations hindering the integration of AI in GCD nanotoxicity assessment. This diagram outlines the key obstacles that must be addressed for the successful adoption of AI in this field, including data scarcity and quality, model interoperability, poor generalizability, multi scale data integration, and a lack of standardization and validation.

While there have been significant advancements in the use of AI models in nanotoxicity evaluations, several critical limitations and challenges currently impede their widespread adoption and full potential realization as summarized in Figure 7. A primary challenge is data scarcity and quality, as the lack of large-scale, standardized datasets constrain s model generalizability and performance (Forest, 2022; del Giudice et al., 2024; Zhou et al., 2024). Secondly, the black box nature of many AI algorithms impairs model interpretability, making it difficult to elucidate the underlying toxicological mechanisms, eroding confidence among researchers and regulators (Jia et al., 2023; Kleinstreuer and Hartung, 2024). Consequently, AI models often struggle with generalizability to novel nanomaterials not represented in their training sets (Hosni et al., 2025). Furthermore, integrating multi scale data—from atomic properties to organismal responses—into a coherent predictive framework presents a significant challenge (Singh et al., 2023). Finally, there remains a critical need for rigorous experimental validation and greater standardization of protocols to substantiate AI-derived insights and ensure the reliability and reproducibility of findings (Yan et al., 2023).

Overcoming these multifaceted challenges demands interdisciplinary collaboration, effective data sharing, and a dedicated focus on developing interpretable AI methodologies. Such efforts are crucial for making AI-driven nanotoxicity assessments more robust, reliable, and practically applicable in biomedicine.

## 4. Intelligent drug delivery from an AI- assisted GCDs perspective

**Figure 8:**
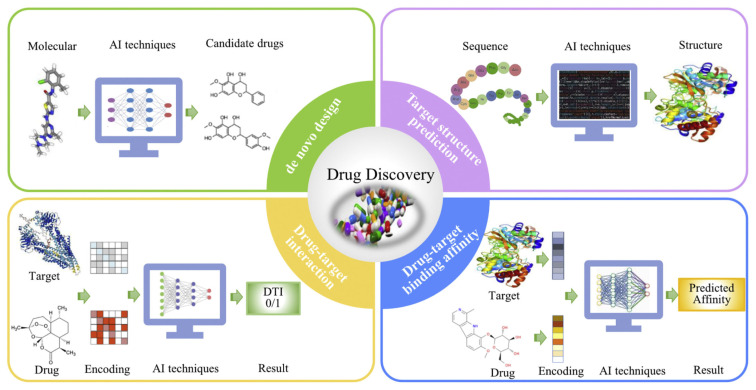
AI applications in natural product-inspired drug discovery, including de novo design, target structure prediction, drug-target interaction modeling, and binding affinity estimation (Chen et al., 2023).

GCDs have emerged as highly promising nanocarriers for drug delivery due to their ultra small size, large surface area, inherent biocompatibility, and photoluminescent properties (Mansuriya and Altintas, 2021; Li et al., 2023). These features enable GCDs to traverse physiological barriers, selectively accumulate in target tissues, and facilitate real-time imaging during therapeutic interventions. Unlike conventional drug carriers, GCDs offer highly tunable surface functionalities that can be precisely engineered to enhance drug loading efficiency, enable stimuli-responsive release, and improve biodistribution profiles (Xu et al., 2024). Nevertheless, the rational development of GCD-based drug delivery platforms increasingly relies on the integration of experimental findings with AI-guided predictive modeling to accelerate optimization and ensure reproducibility. Figure 8 exemplifies how AI technologies are transforming various stages of drug discovery—from de novo compound design to drug– target interaction prediction—highlighting their potential synergy with GCD-based systems for next-generation therapeutics (Chen et al., 2023).

### 4.1. AI- driven drug delivery design

AI offers a transformative paradigm for the design of drug delivery systems, particularly through its ability to handle high-dimensional, nonlinear datasets encompassing physicochemical parameters, biological interactions, and therapeutic outcomes. In the context of GCDs, AI-guided workflows enable systematic formulation optimization, from precursor selection to post synthetic surface modification (Gao et al., 2024b).

The integration of AI into in silico modeling pipelines has significantly improved the predictive accuracy of drug–nanocarrier interactions, particularly for systems involving carbon-based nanomaterials such as GCDs. Traditional approaches relying on molecular docking or quantitative structure - activity relationship (QSAR) methods are increasingly being replaced or augmented by AI-driven strategies that combine structural modeling with data-driven learning (Odugbemi et al., 2024).

Molecular docking simulations are used to estimate the binding affinity and pose stability between nanocarrier surfaces and drug molecules. However, conventional scoring functions often fail to capture the complexity of non covalent interactions specific to GCDs, such as π–π stacking or surface polarity effects (Wang et al., 2014; Novikov, 2023; Pan et al., 2025). Recent advances have leveraged ML to develop data-driven scoring functions that precisely capture complex molecular interactions, thereby significantly enhancing docking precision. For instance, Ji et al. (2023) developed an AI-enhanced docking workflow to model drug interactions with nitrogen-doped carbon nanomaterials, integrating electrostatic descriptors with DL models to predict binding free energy . Their results showed improved discrimination between high- and low-affinity ligands, particularly for π-stacking and hydrogen bonding interactions critical to carbon-based nanocarriers. Such AI-enhanced scoring systems are increasingly adapted to nanocarrier–drug interfaces to better model carbon-based interaction geometries.

The robustness and predictive power of QSAR modeling can be significantly enhanced when integrated with AI algorithms. The ability of AI to discern complex, non linear relationships, manage vast datasets, and autonomously extract intricate molecular patterns profoundly augments the traditional analytical scope of QSAR (Ye and Ouyang, 2024). In AI-driven drug delivery design, QSAR applications, often using advanced regression techniques like random forests and deep neural networks, are crucial for accurately predicting key drug delivery parameters such as encapsulation efficiency and release kinetics. Tropsha et al. (2024) emphasize that AI-augmented QSAR pipelines must ensure reproducibility, chemical interpretability, and rigorous validation, especially when modeling heterogeneous systems like GCDs. Building on this, Noviandy et al. (2024) tested a transferable classification-based QSAR pipeline using ensembled learning models (LightGBM, XGBoost, and Extra Trees) with molecular descriptors, highlighting the importance of metrics like ROC-AUC in imbalanced datasets. Complementing these, Xin et al. (2024) proposed a dimensionality-optimized QSAR workflow integrating diverse descriptors with recursive feature elimination and principal component analysis (PCA), enhancing model robustness and interpretability for complex, tunable nanocarriers like GCDs.

Optimizing drug loading efficiency and release kinetics is fundamental for nanocarrier design, particularly for GCDs, where surface heterogeneity and nanoscale curvature directly impact encapsulation and diffusion. ML algorithms offer a significant advantage over traditional trial-and-error methods by effectively modeling and predicting these complex parameters. For instance, Gholap et al. (2024) successfully used SVR models with SHAP-based explainability to predict drug loading capacity and sustained release in carbon nanostructures. Their findings highlighted that parameters like aromaticity index, surface polarity, and dipole moment are strong predictors of π– π stacking interactions and diffusion-controlled release, directly informing GCD design due to their similar π-conjugated domains and tunable functional groups. Complementing this, Noorain et al. (2023) introduced a probabilistic GPR approach to model drug diffusion profiles. Although not applied to carbon-based carriers, the GPR model quantif ies predictive uncertainty. This makes it highly suitable for GCD systems, where environmental factors like pH, ionic strength, and surface hydration significantly influence drug elution.

Collectively, these ML strategies represent a paradigm shift from empirical formulation design to predictive and explainable modeling in nanomedicine. By integrating feature selection, model interpretability, and probabilistic learning, these tools offer a robust foundation for engineering GCD-based nanocarriers with tailored loading and release characteristics.

### 4.2. Mechanisms of drug loading and release

Effective drug delivery systems require selecting suitable carriers, overcoming physiological barriers, reducing systemic side effects and enabling targeted release to ensure clinical efficacy (Voronin et al., 2021). Drug loading and release mechanisms are pivotal to therapeutic outcomes (Ulucan-Karnak et al., 2023; Ciftci et al., 2025). Nanomaterials, including GCDs, significantly enhance bioavailability by improving drug solubility, protection, barrier passage, and passive targeting (Patra et al., 2018; Ulucan-Karnak and Kuru, 2023; Zhuo et al., 2024). With their versatile surface chemistry, tunable optics, and intrinsic biocompatibility, GCDs are promising components for next-generation smart drug delivery, contributing to highly efficient and eco friendly therapeutic systems when combined with advanced formulations (Rumondor et al., 2016). There are 2 main ways for incorporating pharmaceuticals into delivery vehicles: encapsulation and covalent attachment.

Encapsulation (non covalent loading), physically entraps drugs within carriers using non covalent interactions, offering simplicity, drug integrity preservation, versatility, and cost effectiveness. However, it can suffer from low loading capacity, potential burst release, and environmental instability (Klojdová et al., 2023; Herdiana et al., 2024; Rojas et al., 2024). Recent studies have highlighted GCD encapsulation versatility. Ghataty et al. (2023) achieved over 85% encapsulation of linezolid in bovine serum albumin CDs for wound healing, while Sun et al. (2020) found 50.82% doxorubicin (DOX) encapsulation with pH-dependent release. Tejwan et al. (2022) also showed sustained release of rutin from Korean ginseng-derived CDs.

Covalent attachment (conjugation) involves chemically bonding drugs to carriers via cleavable links, forming prodrugs that activate at target sites. This approach enhances stability, reduces premature release, improves targeting, and diminishes systemic toxicity, though it requires drug modification, complex synthesis, and can lead to slower release if cleavage is inefficient (Manandhar et al., 2021; Junyaprasert and Thummarati, 2023; Parashar et al., 2024). Dada et al. (2022) exemplified this with folic acid-functionalized CDs nanoparticles (FA-CD NPs) for targeted DOX delivery to cancer cells, showing how noncovalent FA-CD-DOX achieved significant DOX release in acidic conditions, minimizing healthy cell cytotoxicity.

While encapsulation is widely used for GCD-based drug delivery due to its simplicity, covalent attachment is crucial for enhanced stability, sustained release, and precise stimulus-responsive delivery, despite being less explored for GCDs. Stimuli-responsive drug release is particularly relevant for GCDs, leveraging their customizable characteristics and intrinsic biocompatibility to control therapeutic agent release via internal or external cues. This boosts accumulation at target sites, improves bioavailability, and enhances overall efficacy while minimizing off-target effects (Rana et al., 2023).

These stimuli can originate from either the physiological environment or be applied externally. Internal stimuli like pH-responsive release (e.g., acidic tumor microenvironments) and enzyme-responsive systems (utilizing specific enzyme-cleavable bonds) enable precise drug delivery to diseased tissues (Andresen et al., 2010; de la Rica et al., 2012; Zelzer et al., 2012; Hu et al., 2014; Sun et al., 2017; Khan et al., 2020; Köhler et al., 2025; Nayanathara et al., 2025). External stimuli provide non invasive remote control, with examples including light-responsiveness (leveraging the photoluminescence and photothermal/photodynamic effects of GCDs), magnetic responsiveness via magnetic nanoparticles, temperature changes, ultrasound, and electrical stimulation (Grüll and Langereis, 2012; Amin et al., 2020; Etefa and Dejene, 2025). Recent studies have shown how versatile GCDs are. Mathur et al. (2025) developed pH-responsive curcuminoid-derived GCDs for methotrexate delivery. Roy et al. (2020) developed thermoresponsive GCD films for transdermal lidocaine delivery . Zhao et al. (2017) engineered PEI-functionalized GCDs on mesoporous silica nanoparticles for enzyme- and redox-responsive DOX release in tumors, enabling real-time imaging.

Despite these advancements, designing and optimizing GCD-based drug delivery systems remains highly complex and resource-intensive, with current empirical approaches often inefficient given the vast combinatorial space of synthesis parameters, functionalizations, loading methods, and stimuli-responsive triggers. AI offers a transformative solution, capable of revolutionizing the rational design of these systems by predicting optimal loading capacities and release kinetics, accelerating the discovery of novel stimuli-responsive mechanisms . This addresses limitations in current systems like inter patient variability in enzyme activity and optimizing multi stimuli responsive systems. Furthermore, AI can significantly reduce experimental burden and cost by predicting promising candidates and optimizing synthesis protocols, ultimately enabling a more predictive, efficient, and personalized design of GCD-based drug delivery systems to enhance clinical efficacy and patient outcomes.

### 4.3. Personalized medicine applications using predictive modeling

**Figure 9:**
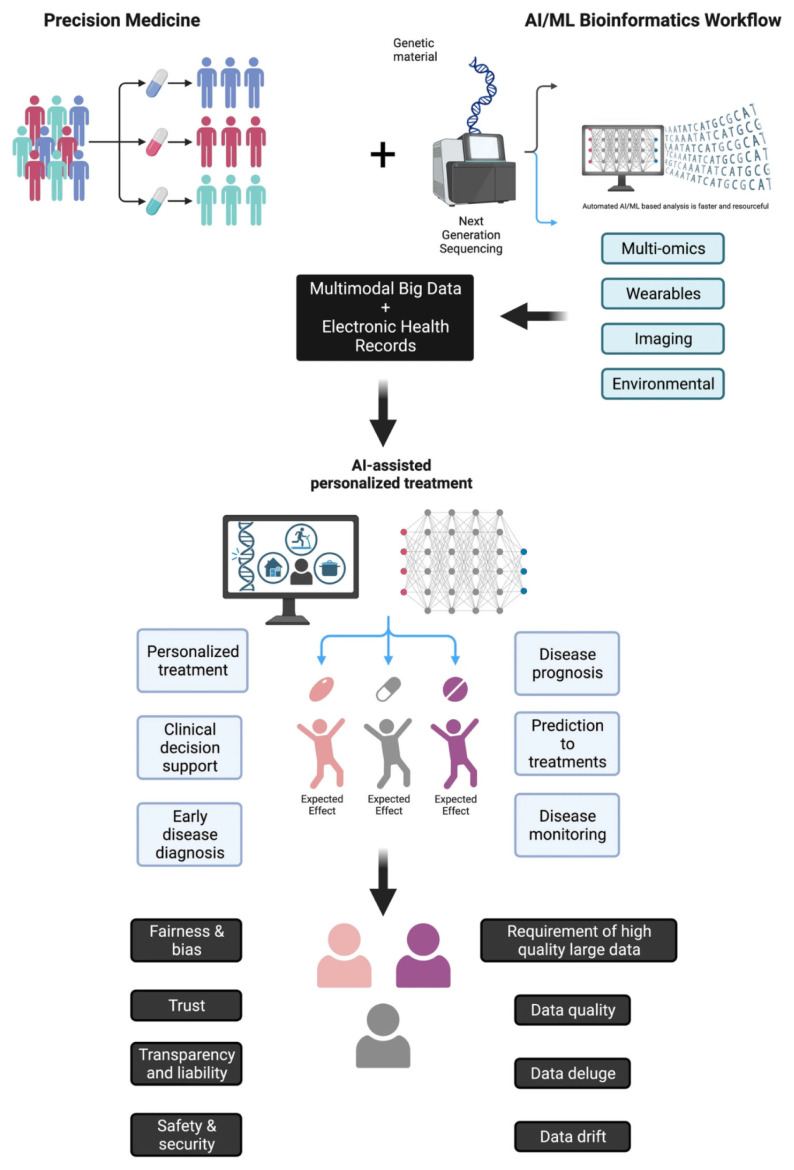
Overview of an AI/ML-driven bioinformatics workflow enabling personalized medicine. Genomic sequencing, electronic health records, multi omics data, and real-time clinical data are processed through AI to deliver tailored treatment strategies. Applications span predictive modeling, disease monitoring, therapy selection, and clinical decision support while addressing data quality and ethical challenges. Adapted from Carini and Seyhan (2024).

Predictive modeling and AI-driven analytics are enabling a shift toward personalized medicine, where nanocarriers such as GCDs, with their tunable chemistry and biocompatibility, offer an ideal platform. These can be integrated with AI models to forecast individual pharmacokinetic and pharmacodynamic behavior. Marques et al. (2024) proposed a hybrid computational pipeline that combines imaging, molecular features, and patient data to predict drug release kinetics and therapeutic outcomes in cancer nanotherapy. This modular approach is adaptable to GCD systems by incorporating GCD-specific descriptors into personalized inference models (Marques et al., 2024). Beyond predictive modeling, AI-enhanced nanomedicine facilitates personalized therapy through patient stratification and dose titration. AI algorithms can analyze patient-specific biomarkers to segment populations, enabling tailored GCD-based therapies that account for individual variations in factors like immunomodulation or ROS sensitivity. A key strategy involves integrating AI into biosensor-feedback loops, allowing for dynamic adjustment of drug administration based on real-time physiological readouts. GCDs, frequently used in sensing, can be embedded in these frameworks for continuous feedback on drug levels, release rates, or inflammatory signals, thereby enabling AI-informed adaptive therapeutic control at the patient-specific level. This systems-level perspective is illustrated in Figure 9, which schematizes the integration of multimodal data and AI/ML pipelines into personalized medicine workflows (Carini and Seyhan, 2024).

From a systems-level perspective, Kothinti (2025) highlighted the necessity of integrating genomic, phenotypic, and environmental data into AI-driven clinical decision tools for ethical and effective personalization. Emphasizing fairness, explainability, and real-time learning, these considerations are highly relevant for GCD-based platforms, where surface functionalization may need to adapt to patient-specific demographic or disease features like enzymatic profiles or metabolic rates (Kothinti, 2025). Collectively, these studies highlight a convergent theme: predictive modeling allows for the adaptation of nanocarriers—especially GCDs—to the unique molecular and clinical characteristics of individual patients. The synergy among AI interpretability, patient-level biometrics, and GCD surface engineering creates a tangible pathway toward intelligent, personalized, and dynamically adjustable therapeutic platforms.

### 4.4. Case studies and emerging therapies

GCDs are multifunctional nanocarriers with immense promise across diverse biomedical applications, including drug delivery, diagnostics, and bioimaging, due to their biocompatibility, fluorescence tunability, and surface versatility. In oncology, GCDs are being explored for site-specific drug release and real-time imaging in photothermal therapy (PTT), with ML algorithms (e.g., gradient-boosted decision trees (GBDTs) and CNNs) being integrated to fine-tune formulations for precise heat generation and enhanced tumor specificity (Agboklu et al., 2024). However, challenges like tumor heterogeneity and off-target accumulation persist. In antimicrobial applications, naturally derived GCDs have broad-spectrum activity via photo dynamically induced ROS generation, enhancing antimicrobial agent efficacy (Parvin et al., 2025). However, consistent efficiency across strains and host microbiota interference remains a concern.

For inflammation modulation, ROS-sensitive GCDs are used for targeted NSAID delivery in oxidative microenvironments, offering concurrent diagnostic capabilities (Etefa and Dejene, 2025); yet, variations in ROS levels and stability under oxidative stress require further investigation. Finally, hybrid GCD designs, such as silica-GCD nanohybrids, integrate structural stability with bioresponsive properties for robust drug delivery, despite ongoing concerns regarding interfacial stability and scalability. These diverse applications highlight the significant potential of GCDs, especially when integrated with AI, to address complex biomedical challenges, though further validation and optimization are crucial for successful clinical translation (Liu et al., 2024).

### 4.5. Clinical translation and challenges

Despite their therapeutic promise, GCD-based nanocarriers face significant hurdles in clinical translation. A major issue is scalability and batch-to-batch consistency, as current production methods often fail to meet good manufacturing practices (GMP) requirements, thereby hindering reproducibility and quality assurance (Hulme, 2022). Furthermore, GCDs occupy a regulatory gra y zone. Unlike established drug delivery systems, there are no dedicated approval pathways, and regulatory bodies have not fully addressed their long-term safety, biodistribution, or clearance. Insufficient standardization in physicochemical characterization, like zeta potential and QY, further complicates regulatory evaluation (Tripathy et al., 2025).

Another limitation lies in in vivo complexity. While GCDs are often heralded for their low cytotoxicity and intrinsic fluorescence, their interaction with biological fluids and immune recognition pathways is not yet fully understood. The absence of harmonized preclinical protocols further complicates translational consistency across laboratories and regions. Nonetheless, the growing body of literature suggests a clear translational trajectory. Sheikh and Jirvankar (2024) showed that AI models—such as GBDTs and deep neural networks—can predict nanoparticle characteristics (e.g., size distribution and surface ligand density) and flag batch inconsistencies before scale-up production, mitigating scalability and regulatory hurdles.

**Figure 10:**
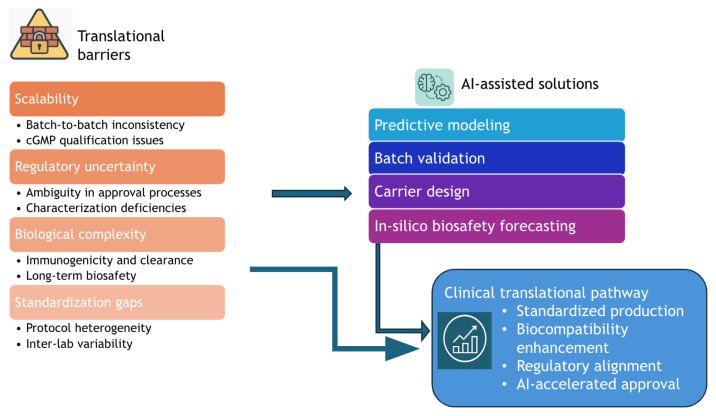
Overcoming translational barriers for GCD nanocarriers with AI- powered solutions. This figure presents a strategic roadmap showing how AI can be used to address key challenges like scalability, regulatory uncertainty, and standardization gaps, ultimately accelerating the pathway toward clinical application.

GCDs show immense promise as nanocarriers for treating cancer, and infectious and inflammatory diseases. However, their clinical translation faces significant hurdles, including scalability, regulatory uncertainty, patient variability, and unclear long-term biosafety. As Figure 10 illustrates, future research must bridge the gap between preclinical innovation and clinical viability. This requires standardizing production, improving biocompatibility, and leveraging AI for predictive modeling of drug release, as well as generating novel nanocarrier structures. A synergistic approach, combining molecular design, advanced computation, and rigorous clinical insight, will be crucial for developing safe and effective GCD-based therapies.

## 5. Advanced diagnostics

GCD-based advanced diagnostics leverage the unique optical and physicochemical properties of GCDs to deliver highly sensitive, specific, and rapid detection beyond the capabilities of conventional methods, thereby revolutionizing biosensing and disease diagnosis.

### 5.1. GCDs in biosensing and detection

GCDs are highly promising for biosensing and diagnostic applications due to their economical, eco friendly production, intrinsic photoluminescence, high biocompatibility, low toxicity, tunable optical properties, and abundant surface functional groups. These attributes make them potent fluorescent probes, utilizing diverse detection mechanisms such as fluorescence enhancement (e.g., analyte-induced aggregation/disaggregation, conformational changes) and fluorescence quenching (e.g., fluorescence resonance energy transfer , photoinduced electron transfer, static quenching, internal filtering effect) (Agrawal et al., 2021; Surana and Bhattacharya, 2021; Dai et al., 2022; Tian et al., 2023; Shang et al., 2024; Lodha et al., 2024). This versatility allows for sensitive and specific identification of biomolecules, environmental pollutants, and medical indicators, with their green nature further enhancing their appeal for sustainable biomedical production. (Agrawal et al., 2021; Surana and Bhattacharya, 2021; Dai et al., 2022; Tian et al., 2023; Shang et al., 2024; Lodha et al., 2024).

Ahn et al. (2019) developed nitrogen-doped carbon dots (N-CDs) from hamburger sandwich waste for selective Fe^3+^ sensing and bioimaging. Tafreshi et al. (2020) used cauliflower juice-derived CDs for high-selectivity pesticide detection. Bavya et al. (2025) synthesized sulfur-doped CDs from waste bamboo, creating a unique sensor with an increase in fluorescence with mercury ions. Enyoh et al. (2025) utilized fluorescent carbon quantum dots from waste polyethylene terephthalate as dual-mode probes for ciprofloxacin, palladium ions, and fluoxetine, with applications for broad environmental monitoring. Notably, Li et al. (2025) developed a sensor array using 3 herb-derived N-CDs that, when combined with an ML model, enabled rapid, precise, and interference-immune discrimination of warfarin from its metabolites, even in unknown samples.

Despite their versatility, analyzing complex, noisy signals from multi analyte detection or real-world samples presents significant challenges for GCD biosensors. Manual interpretation is time consuming, prone to error, and lacks throughput. AI is crucial here, addressing these limitations by allowing GCD-based biosensing to transcend current capabilities. AI, particularly ML models, can discriminate subtle signal variations, handle complex multi analyte detection, mitigate interference for enhanced selectivity, and enable rapid, real-time analysis. Moreover, AI can predict optimal sensor design, accelerating research and development by analyzing correlations between GCD properties and sensing performance. This unique blend of sustainable synthesis, tunable optics, and versatile sensing, critically augmented by AI, positions GCDs as a powerful solution for analytical chemistry, environmental monitoring, and diagnostics (Jin et al., 2020; Duman and Jalilov, 2024; Ratre et al., 2024). This includes their efficacy in label-free biosensing for macromolecules like proteins. For example, Miao et al. (2016) reported tomato juice-derived CDs for carcinoembryonic antigen detection. Ultimately, AI processes high-dimensional signal data to extract meaningful biological information with high accuracy, maximizing the diagnostic potential of GCDs.

### 5.2. AI- enhanced diagnostic platforms

**Figure 11:**
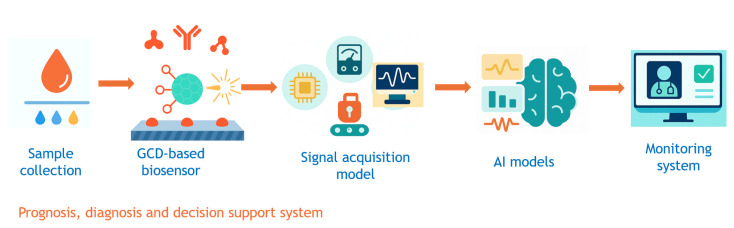
Integrated AI-assisted diagnostic pipeline using GCD-based biosensors. This diagram presents a streamlined process for a smart diagnostic system. It shows how data from a GCD biosensor is processed by AI models to deliver real-time insights for clinical decision support.

The growing convergence of nanotechnology and AI is enabling next-generation diagnostic systems that are portable, responsive, and context aware. GCDs, with their high surface functionality, photostability, and biocompatibility, are excellent candidates for real-time biosensing. However, the non linear, multidimensional, and context-dependent nature of signals from GCD-based sensors necessitates the application of AI-driven analysis frameworks to ensure interpretability and accuracy, as shown in Figure 11. Rather than contributing to sensor design, early applications of ML in biosensing have strategically been positioned to decode and refine the multidimensional signal dynamics inherent to complex bioanalytical environments, thereby paving the way for more precise observations and more reliable measurements.

The output signals from GCD biosensors are inherently complex, often characterized by significant noise and non-Gaussian distributions (Lou et al., 2025). These characteristics primarily stem from the excitation-wavelength-dependent photoluminescence of GCDs and their susceptibility to environmental quenching effects (Goumas et al., 2025). Hatimuria et al. (2023) highlighted that while GCD-modified electrochemical sensors are sensitive, they face signal drift and cross-reactivity in clinical samples, necessitating robust pattern recognition algorithms like support vector regression and CNNs. Addressing clinical heterogeneity, Kang et al. (2024) combined GCD sensors with CRISPR/Cas-based specificity and DL to resolve ambiguous signals. However, their work also showed increased false-positive rates in multiplexed assays without proper AI calibration, indicating challenges in model generalization for real-world settings. The potential for mobile health (mHealth) applications is also significant, as shown by Cui et al. (2022), who developed a smartphone-based system with GCD-functionalized test strips and an embedded decision-tree classifier for rapid contaminant detection, despite variability issues from lighting and user handling . Furthermore, Adil et al. (2023) developed multi modal GCD biosensing platforms that combine fluorescence and electrochemical data, where data fusion improved diagnostic confidence. They identified a critical need for standardized, multi modal training datasets as generalizing unimodal AI models remains a challenge.

Despite the transformative potential of AI in GCD diagnostics, significant limitations persist, including data scarcity, lack of benchmarking protocols, and insufficient model explainability. This hinders clinical trust and regulatory approval. GCD signal heterogeneity across batches also challenges current ML models. Future research must prioritize developing cross-modal training datasets and implementing interpretable AI modules like SHAP for transparent diagnostic workflows. Establishing GMP-grade GCD production protocols is essential for reproducibility, alongside federated learning for privacy-preserving updates in mHealth systems. Realizing the promise of GCDs for real-time, AI-integrated diagnostics demands a deeply interdisciplinary approach spanning nanomaterials, ML, clinical diagnostics, and regulatory science to ensure their translation into robust tools for point-of-care and personalized healthcare.

### 5.3. Multiplexed and multi modal detection

The simultaneous detection of multiple biomarkers using GCDs is a significant advancement for diagnostics and personalized medicine. Tunable fluorescence in GCDs enables multiplexed detection systems, such as the multicolor biosensor developed by Tong et al. (2022). Integrated into a laser-printed paper-based microfluidic chip, this system allowed cost-effective, simultaneous detection of 3 targets, highlighting spectral versatility and translational potential. The need for real-time, high-throughput screening has driven the application of ML algorithms (Revelou et al., 2025). Ram et al. (2024) highlighted the role of DL in interpreting complex optical outputs from GCDs, especially in microfluidic environments. DL was essential for noise filtering, dynamic range expansion, and feature classification, particularly with non linear kinetics or overlapping spectral signals .

Despite these promising advancements, significant challenges continue to impede the widespread application of GCDs in multiplexed detection. For instance, batch-to-batch inconsistency in critical GCD parameters—including size, surface functionalization, and emission stability—has been reported to compromise the reliability and reproducibility of multiplexed assays (Liu et al., 2024; Wang et al., 2024). Furthermore, while the use of multicolor GCDs can address issues of spectral overlap, Mohammadzadeh kakhki and Mohammadpoor (2024) noted that the absence of rigorous calibration and standardized data processing protocols remains a formidable obstacle, particularly in low-resource settings. Lu et al. (2019) describes the synthesis of highly fluorescent, N-CDs that have striking blue luminescence upon excitation with both UV (378 nm) and near-infrared (750 nm) light, and had highly sensitive and selective multimodal detection (both down- and upconversion) specifically for mercury ions (Hg^2+^). To pass these limitations, data fusion strategies that combine optical, electrochemical, and thermal sensing modalities have been proposed as a viable solution.

Future research should focus on enhancing spectral resolution, developing scalable and uniform GCD fabrication methods, and establishing robust AI-assisted calibration frameworks for multi signal interpretation. A multidisciplinary approach combining materials science, microfluidics, data science, and clinical diagnostics is essential to translate these multiplexed biosensing platforms from concept to widespread clinical use.

## 6. Bioimaging applications

The emergence of GCDs represents a paradigm shift for modern bioimaging, owing to their exceptional photostability, tunable luminescence, and high biocompatibility (Kar et al., 2024). However, the deployment of these probes in advanced microscopy techniques—such as 3D confocal imaging and high-content, time-lapse studies—yields datasets of vast scale and intricacy. This data deluge precludes effective manual analysis, thus hindering the translation of rich visual information into robust, quantitative insights. This is why DL and computer vision frameworks become critical (Mao and He, 2024). By automatically segmenting, classifying, and tracking features within these complex image sets, AI enables the extraction of reproducible and salient biological patterns that would otherwise be imperceptible. Accordingly, this section will elucidate how the superior optical signals provided by GCDs are synergistically leveraged by AI to power new frontiers in biological discovery.

### 6.1. Imaging modalities

GCDs are at the forefront of modern bioimaging, particularly in fluorescence imaging and with growing promise in approaches like photoacoustic imaging. Their fundamental advantages include low cytotoxicity, high QY (bright fluorescence), and photostability, enabling clear, sensitive, and long-term biological monitoring compared to heavy metal-based alternatives (Das et al., 2023; Shang et al., 2024).

Fluorescence imaging is the most common application for GCDs, with their tunable emission wavelengths making them versatile probes for cellular and in vivo experiments. Examples include bitter orange juice-derived GCDs showing vivid, pH-dependent fluorescence for imaging SKBR3 and NIH/3T3 cells with high cell viability (Fatahi et al., 2019). Highly luminous, biocompatible CDs from potato enabled multicolor imaging of HeLa cells without cytotoxicity (Mehta et al., 2014). Furthermore, orange juice-derived carbon quantum dots (CQDs) formed CQD/Ag heterostructures that give superior imaging and induction of cell death in human colorectal cancer cells (Mishra et al., 2023b). Beyond typical fluorescence, GCDs have been explored in photoacoustic imaging, leveraging their near-infrared absorption. Honey-derived carbon nanoparticles, for instance, served as excellent exogenous agents for in vivo lymph node imaging in murine models, producing significant photoacoustic signals (Wu et al., 2013).

Integrating high-resolution fluorescence with deeper-penetrating photoacoustic tomography offers prospects for multimodal imaging. This aligns with the increasing necessity for AI-driven image analysis. While still in early stages, AI integration provides a robust foundation for future AI-enhanced biomedical imaging techniques using GCDs. Without AI, the manual analysis of multimodal bioimaging data becomes immensely challenging, leading to time-consuming processes, potential human error, and an inability to fully extract subtle, complex patterns. AI can integrate multimodal datasets, significantly improve signal-to-noise classification, and identify subtle patterns in biodistribution or diseased tissues that are imperceptible to the human eye. This enables more precise observations, enhances diagnostic accuracy, and facilitates the translation of GCDs into advanced, real-time, and high-throughput bioimaging platforms.

### 6.2 Deep learning and computer vision in bioimaging

The synergy of GCDs with DL and computer vision has revolutionized bioimaging, enabling high-resolution, real-time, and label-specific visualization of cellular events. GCDs, with their tunable emission, biocompatibility, and photostability, act as effective contrast agents, providing the robust optical signals crucial for accurate DL model interpretation (Shang et al., 2024; Etefa and Dejene, 2025).

DL is increasingly vital for segmentation and analysis of CD-labeled structures. Kannouma et al. (2024) highlighted the role of GCDs in selective organelle staining. DL was critical for automated delineation and for optimizing GCDs in bioimaging. Complementing this, Anaam et al. (2023) showcased a post segmentation pipeline using a deep generative adversarial network for dataset augmentation and a self-attention CNN for cell classification, showing how DL can be useful for probe design and biological analysis. For object detection tasks, CNNs and transformer-based vision models have enabled precise localization of microstructural features. Agarwal and Chumbhale (2025) utilized GCDs in brain tissue sections and trained a ML model for early-stage neuroblastoma detection^2^ . Sarmanova et al. (2022) applied v ision transformers to GCD-labeled fluorescence maps of organoids, achieving superior detection accuracy in low-intensity datasets . Moreover, Wang et al. (2023) combined CNN-based detection with SHAP interpretability to visualize critical image regions influencing AI decisions in tumor diagnostics. There have also been recent advancements in GCD-assisted DL in high-resolution modalities. Huo et al. (2024) used carbonized polymer dots for super resolution microscopy of lipid droplets, integrating segmentation pipelines with attention-guided feature extractors for sub organelle analysis. Patyal et al. (2025) extended this to mitochondrial imaging using GCDs under live-cell conditions, leveraging ResNet backbones for dynamic event tracking in time-lapse sequences . The development of bimodal imaging platforms has also been facilitated by DL techniques. Zeng et al. (2023) reported a tumor-microenvironment-activated bimodal probe (fluorescence and photoacoustic) using carbon nanodots, where a DL-assisted signal fusion model was trained to classify tumor margins based on multimodal datasets. Mao and He (2024) showed that hybrid CNN-RNN models could predict temporal dynamics of GCD uptake in glioblastoma models, guiding AI-augmented bioimaging protocols for drug targeting . Despite these promising developments, several limitations persist. Jiang et al. (2024) emphasized the lack of standardized image datasets for GCD-labeled tissues, which hinders model reproducibility and benchmarking. Anaam et al. (2023) also cautioned against overfitting in GCD-based bioimaging models due to high signal redundancy and unbalanced class distributions. Moreover, while SHAP and attention-based visualizations provide some interpretability, they often lack consistency across model architectures, especially in multimodal imaging workflows (Anaam et al., 2023).

Compared to traditional markers, GCDs offer superior photostability and biocompatibility for prolonged live imaging. However, their optical heterogeneity across synthesis batches necessitates adaptive DL models for domain generalization. While CNNs remain primary for spatial tasks, transformer-based models show promise for long-range spatial dependencies in low-signal environments. Future research should focus on creating open-access, high-resolution datasets of GCD-labeled tissues. Incorporating hybrid DL models (CNNs, transformers and temporal architectures) can facilitate multimodal image understanding. Integrating XAI frameworks is crucial for clinical trust. Ultimately, interdisciplinary collaboration among nanotechnologists, AI experts, and clinicians is vital to design robust, generalizable, and ethically sound GCD-based diagnostic tools.

### 6.3 Theranostics: dual diagnostic and therapeutic platforms

GCDs present a versatile platform for theranostics, enabling simultaneous real-time imaging and drug delivery, crucial for precision medicine (Fathi-karkan et al., 2025). Their intrinsic fluorescence allows non invasive tracking of therapeutic distribution and release. Wang et al. (2017b) tested the use of GCDs in dual-responsive nanocarriers for tumor-specific DOX release, with real-time fluorescent feedback on cellular uptake. These systems, combined with advancing AI-assisted image analysis, pave the way for intelligent, feedback-controlled nanomedicine. GCDs are well-suited for theranostic applications due to their photostability, biocompatibility, and surface tunability, making them effective in both imaging (e.g., fluorescence and MRI) and therapeutic strategies like PTT, photodynamic (PDT), and chemotherapy (Zhong et al., 2023; Wang et al., 2024). A key feature is their responsiveness to tumor microenvironment cues (e.g., low pH and ROS). Wu et al. (2021) developed a GCD composite for acidic environment-specific drug release with simultaneous imaging and Chen et al. (2021) developed a pH -triggered drug release system. However, precise control in vivo remains challenging (Chen et al., 2021). Surface functionalization in moieties that enhance tumor accumulation and reduc e off-target effects is critical (Sengar et al., 2022).

**Figure 12:**
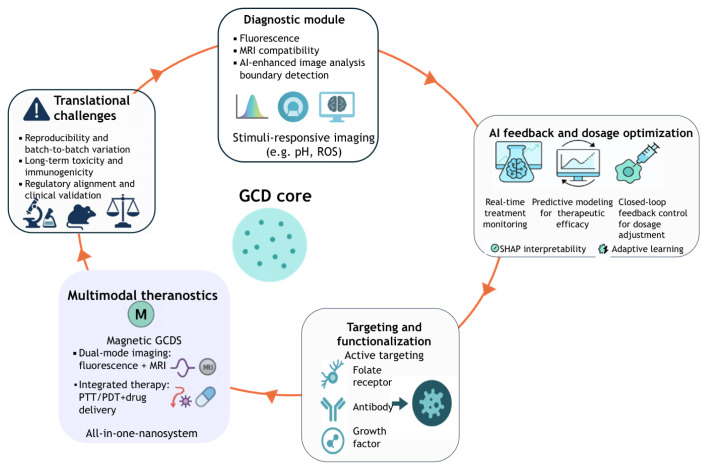
Schematic overview of an AI-enabled theranostic platform utilizing GCDs. This figure illustrates a closed-loop system where GCDs serve as a core component for a multimodal theranostic nanoplatform. It outlines the key modules, including diagnostic and therapeutic modules, and their integration with targeting a functionalization for enhanced specificity. The system is further optimized by an AI feedback and dosage optimization loop that enables real-time monitoring and predictive modeling. Finally, it addresses critical translational challenges and highlights the potential for multimodal theranostics, showcasing GCDs as an all-in-one nanosystem.

AI significantly advances theranostics by enabling real-time monitoring and predictive modeling. Hussain et al. (2023) highlighted the use of AI-enhanced DL frameworks to process GCD-based imaging data, allowing for accurate tumor boundary detection and treatment response prediction. Li (2023) proposed AI-assisted closed-loop feedback for dosage optimization. These powerful frameworks, however, demand harmonized datasets and regulatory-standard algorithms (Li et al., 2023). A notable theranostic paradigm is the integration of multimodal imaging and therapy in a single GCD-based platform. Zhong et al. (2023) introduced magnetic GCDs that combine fluorescence and MR imaging with heat-mediated therapeutic output, exemplifying a truly multifunctional system. However, scalability, material consistency, and long-term toxicity remain hurdles for clinical translation (Wang et al., 2024). A schematic representation of such an intelligent, modular GCD-based theranostic system is provided in Figure 12, highlighting the integration of diagnostics, targeted therapy, AI-driven feedback, and translational challenges.

In summary, while GCD-based theranostics are versatile platforms, limitations persist. Stimuli-responsive mechanisms lack consistency in vivo, surface functionalization introduces complexity and immunogenicity, and AI integration faces standardization challenges. Multimodal systems, despite being cutting edge, are far from ready for mass production. Future research must establish unified efficacy protocols, expand AI training datasets, design scalable GMP-compliant synthesis routes, and foster a multidisciplinary approach to translate GCD-based theranostics into routine clinical application.

## 7. AI and ML integration for GCDs

The strategic integration of AI and ML is revolutionizing data generation and management, and is driving breakthroughs in predictive modeling and automation. It is thus vital for advancing GCD applications.

### 7.1. Data generation and management

The successful integration of AI into GCD-based biomedical research hinges on the availability of well-structured, high-quality datasets. Currently, data fragmentation and inconsistencies in reporting biocompatibility, toxicity, imaging modalities, and synthesis conditions impede progress. To address these challenges, recent efforts such as OptiDots_v1.0 highlight the potential for centralized data curation that supports ML workflows (Goularte dos Santos et al., 2025). OptiDots_v1.0a is a curated database encompassing 199 GCD samples with metadata on synthesis parameters (e.g., time, temperature, and precursor), particle size, QY, and fluorescence properties Adopting FAIR data principles—making datasets findable, accessible, interoperable, and reusable—is increasingly recognized as essential in nanomaterials research (Serrano et al., 2024). NanoCommons infrastructure, for instance, provides an operational framework to make nanosafety data FAIR, including toxicity and physicochemical metadata, enhancing data reuse for predictive modeling (Wierling et al., 2021). Crucially, such standardized and FAIR-compliant data infrastructures directly address the critical challenge of batch-to-batch inconsistency (as discussed in section 8.1) by enhancing the comparability and reproducibility of synthesis protocols across different research groups.

Robust data preprocessing is vital for GCD research. This includes background subtraction, spectral unmixing, and normalization for fluorescence microscopy datasets to minimize variability. For classification, data augmentation (e.g., synthetic minority oversampling technique, generative adversarial networks ) helps with class imbalance and overfitting. Feature engineering is also crucial; beyond pixel-level features, physicochemical descriptors (e.g., zeta potential and size distribution) must be encoded (Mujahid et al., 2024). Dimensionality reduction methods like principal component analysis (PCA) and t-distributed stochastic neighbor embedding (t-SNE) effectively visualize feature clusters and correlations between GCD characteristics and biological responses. Ultimately, building interoperable, metadata-rich databases, supported by semantic standards and harmonized preprocessing, forms the foundation for scaling AI-driven GCD research (Choudhary et al., 2022). Platforms like NanoCommons, coupled with strong metadata stewardship, are key enablers for predictive, reproducible, and clinically translational nanomedicine (Serrano et al., 2024).

### 7.2. Predictive modeling and automation

The integration of predictive modeling and automation is transforming GCD research by optimizing experimental design and enhancing scalability (Nolte and Tomforde, 2025). Reinforcement learning (RL) and Bayesian optimization BO) are key tools for adaptive experimentation, enabling rapid, low-intervention identification of optimal synthesis conditions. RL allows agents to iteratively improve synthesis yields based on real-time feedback, as seen in nanomaterial synthesis workflows where reward functions are tied to metrics like QY (Tavernaro et al., 2024). BO, conversely, excels in low-data regimes, autonomously exploring high-dimensional synthesis spaces and uncovering new mechanistic insights. Rao and Grzelczak (2024) used BO to optimize El-Sayed-type synthes is . These techniques are often coupled with active learning pipelines to prioritize informative experiments and minimize redundant sampling (Kuznetsova et al., 2024; Masud et al., 2024).

Parallel to algorithmic advancements, robotic laboratories are enabling full-loop automation in GCD research. Closed-loop platforms, equipped with in-line characterization and real-time ML-driven decision engines, can autonomously synthesize and test GCDs across vast parameter spaces. Such systems significantly accelerate material discovery, outperforming manual protocols in both throughput and optimization accuracy (Bennett and Abolhasani, 2022). These automated platforms not only optimize existing parameters but are foundational for enabling inverse design, where the high-quality, systematic data generated serves as the essential training ground for generative AI models capable of proposing entirely novel GCD structures and synthesis pathways based on desired therapeutic endpoints.

These approaches offer benefits in speed, reproducibility, and the generation of standardized, high-quality datasets for next-generation AI models. The future of nanomaterial discovery will likely involve hybrid frameworks combining simulation, experimentation, and ML-guided automation.

Despite the paradigm shift, challenges persist. The interpretability of AI model decisions in dynamic synthesis environments is often limited, highlighting the need for explainable ML models that provide mechanistic insight (Saarela and Podgorelec, 2024). Scaling these systems to more complex GCD architectures also requires further innovation in hardware and software. Future work should prioritize open, interoperable platforms integrating robotic synthesis, high-throughput characterization, and AI-driven decision engines, designed for modularity and cross-laboratory transferability. Coupling these within silico prediction frameworks, such as generative models, could drastically reduce experimental burden and uncover novel GCD designs.

### 7.3. Bridging the gap from prediction to scientific discovery

The integration of AI within GCD research faces various difficulties that surpass algorithmic details. At the heart of this issue is a notable disciplinary gap between materials science and data science. This often manifests through disjointed datasets, inconsistent methodologies in reporting, and a deficiency in the meaningful correlation between empirical findings and their computational analogs. While materials scientists possess extensive expertise in reaction mechanisms and structure– property correlations, many lack formal knowledge in data organization, feature extraction, or the level of abstraction that ML techniques necessitate (Greener et al., 2022). Conversely, AI specialists, despite their adeptness in modeling and optimization, frequently find themselves challenged to interpret the underlying physical chemistry reflected in the data, struggling to differentiate between signal and noise or to correlate numerical trends with mechanistic relevance.

This gap amplifies the familiar black box dilemma that is inherent to ML models. The uncertainty associated with these models stems not only from their complex frameworks but also from the lack of chemically informed constraints during the training phase. In the absence of embedded physical insights or domain-specific priors, AI operates merely as an advanced curve-fitting mechanism, capable of forecasting outcomes based on inputs yet incapable of elucidating the reasons for these outcomes or their implications concerning the fundamental chemistry. For example, when temperature is regarded merely as a numerical variable, its influence on modulating carbonization kinetics or its effect on fluorophore stability is overlooked. Within this context-free framework, the transformative potential of AI is diminished to mere incremental enhancements of processes.

Confronting these challenges necessitates the establishment of a robust, machine-readable data framework tailored specifically for GCD systems. Such an ontology ought to unambiguously delineate how experimental variables, such as precursor identity, reaction conditions, purification processes, and QY assessments, are recorded and disseminated. The field of polymer informatics illustrates that standardized and curated datasets can significantly enhance both reproducibility and the reliability of models (Butler et al., 2018; Ge et al., 2025). The advancement of interdisciplinary teamwork is just as crucial. Data scientists and ML experts need to be incorporated into laboratory processes (Sezer et al., 2025; Dokuzparmak et al., 2025). Materials researchers would gain a robust understanding of data organization, algorithmic logic, and computational modeling. These collaborations would support a new generation of researchers capable of thinking in both chemical and computational terms.

The future of AI in this domain may well depend on our ability to build models that do not just learn from data, but are guided by the underlying physics and chemistry. The application of concepts like the Arrhenius equation into modeling structures has shown promise, such as in forecasting ionic conductivity in polymer electrolytes. Such methodologies not only improve model efficiency but also generate outputs that are more comprehensible and less dependent on extensive data sources (Bradford et al., 2023). The application of analogous techniques to GCD research could transform the role of AI from that of a predictive surrogate to a legitimate instrument for scientific inquiry. Through the establishment of standardized data, interdisciplinary fluency, and physically informed modeling, GCD research may evolve from superficial pattern recognition to hypothesis-driven, mechanism-aware investigation (Junaid, 2025).

## 8. Challenges, regulatory aspects, and future perspectives

Navigating the landscape of GCDs for real-world applications necessitates a thorough examination of technical and scientific challenges, crucial regulatory aspects, and critical considerations for commercialization, market outlook, and future directions.

### 8.1. Technical and scientific challenges

Despite promising in vitro results showing low acute cytotoxicity, the long-term toxicity and biodegradability of GCDs remain significant concerns. More research is needed to assess their effects on aquatic species and potential accumulation in vital organs after prolonged exposure (Chung et al., 2021; Hatimuria et al., 2023; Bodele et al., 2025). GCDs also have varied immunomodulatory activities depending on their functionalization; for instance, Ayaz et al. (2020) reported that PEG-passivated CDs significantly reduced IL-6 production in LPS-stimulated macrophages, showing an anti inflammatory effect. Designing biodegradable GCDs and understanding their surface chemistry are crucial to reduce immunological responses, enhance bodily excretion, and ensure safety for repeated or extended use, which is vital for clinical translation and regulatory approval (Jing et al., 2025).

A major challenge stems from the green synthesis of GCDs. Despite using diverse biomass sources and techniques, synthesis introduces significant batch-to-batch variability, hindering consistent biological and physicochemical properties. To ensure repeatability for reliable intelligent drug delivery systems, standardizing synthesis procedures, precursors, and comprehensive characterization is crucial. This includes rigorous purification, precise control over reaction parameters, thorough reporting, and the development of reference materials and interlaboratory studies (Dhumal et al., 2024). These multifaceted and complex challenges highlight the limitations of traditional trial-and-error approaches, making the adoption of the data-driven optimization and standardization strategies detailed in section 7 not just an opportunity, but a necessity for the advancement of the field.

### 8.2. Challenges in governance: ethics, trust, and regulatory compliance

Despite remarkable advancements in AI-driven diagnostics and nanomedicine, significant challenges persist regarding interpretability, data privacy, and ethical compliance in clinical applications. The black box nature of current DL models undermines transparency and trust in high-stakes medical contexts. To counter this, XAI frameworks like gradient-weighted class activation mapping , LIME , and attention-based visualization are crucial for demystifying DL outputs, thereby enhancing clinical acceptance and reliability . However, interpretability alone is insufficient without stringent adherence to data privacy and ethical frameworks. Medical AI systems, which rely on sensitive patient data, must ensure compliance with regulations such as the Health Insurance Portability and Accountability Act (HIPAA) and General Data Protection Regulation (GDPR) through federated learning, anonymization, and robust cybersecurity (Yadav et al., 2023). Crucially, explainable outputs must avoid inadvertently showing patient-specific features, especially in multi institutional settings.

The commercialization of GCD-AI platforms faces a uniquely hybrid regulatory landscape, where a single product must simultaneously satisfy requirements for both nanomaterial safety (overseen by bodies like the FDA and EMA) and digital health software (governed by frameworks like s oftware as a medical device (SaMD))^3^.

Another major concern is algorithmic bias stemming from imbalanced datasets, which can lead to disparities in diagnostic accuracy across demographic groups. Ethical AI mandates systematic evaluation of such biases and integration of fairness constraints during model development and deployment. Frameworks now emphasize the co development of AI systems with clinicians and ethicists to ensure alignment with medical norms and uphold patient autonomy (Qureshi et al., 2024). In essence, achieving trustworthy and ethically aligned AI in nanomedicine requires a 3-pronged strategy: embedding explainability, enforcing stringent data governance, and incorporating fairness-aware learning with multidisciplinary oversight throughout the AI lifecycle. Addressing these challenges is not merely a technical requirement but a societal imperative for AI-enhanced healthcare.

### 8.3. Commercialization and market outlook

Intellectual property (IP) protection in this hybrid domain is increasingly specialized. With over 340,000 AI-related patents filed globally by 2023^4^, approximately 1 300 patent families (2018–2023) specifically address AI and nanomaterial co design. Protecting GCD-AI innovations therefore requires a composite IP strategy covering both the nanomaterial synthesis and its embedded algorithmic logic.

From a commercial standpoint, market trends strongly support AI-enhanced GCD platforms. The carbon quantum dot market is projected to quadruple to USD 5 billion by 2033 (CAGR approximately15.5%), from USD 1.5 billion in 2024^5^. More generally, carbon nanomaterials are expected to reach USD 24.99 billion by 2029, while the global nanotechnology market surged from USD 79 billion in 2023 to a forecasted USD 332 billion by 2032^6^,^7^. The convergence of AI with nanotechnology is projected to expand from USD 9.3 billion in 2023 to over USD 40 billion by 2031 (approximately 20–21 % CAGR)^8^.

Successful market translation demands close collaboration between academia, industry, and regulators. Partnerships like university–CDMO collaborations and public–private consortia are vital for GMP-compliance and premarket approval. AI monitoring tools can aid regulatory compliance through real-time performance logging, but standard operating procedures require validation across regions. Key challenges include material reproducibility and AI drift. Future pathways involve developing standardized GCD reference materials, AI transparency registries for SaMD audits, and utilizing federated learning for privacy-preserving data sharing. Regulatory sandboxes or pilot approvals could accelerate deployment in specific healthcare settings.

### 8.4. Emerging trends and future directions

The field of GCDs is rapidly advancing, driven by the precise engineering of next-generation GCDs with tunable optical, chemical, and biological functionalities through surface chemistry and heteroatom doping (Li and Gong, 2022). Multicolor emission and ratiometric fluorescence designs are particularly noteworthy for enhancing real-time sensing and imaging (Chen et al., 2024).

A major trend is the convergence of multi omics data, advanced nanomaterials, and AI for personalized healthcare. AI models, trained on diverse omics datasets, can now predict disease states, guide GCD-based sensor selection, and optimize functionalization in silico (Molla and Bitew, 2024; Etefa and Dejene, 2025). This synergy extends to integrating GCDs with biosensing microarrays and AI-enhanced signal processing, paving the way for multiplexed diagnostic tools and personalized medicine platforms where GCD sensors dynamically adapt to patient-specific molecular signatures.

Concurrently, there is a strong emphasis on sustainable and scalable GCD production. Moving beyond traditional solvent-heavy methods, research focuses on biomass-derived carbon sources like agricultural waste for large-scale, low-cost manufacturing (Ren et al., 2024). Photochemical and microwave-assisted synthesis methods are also being optimized for energy efficiency and reproducibility for environmentally responsible production.

Despite ongoing challenges in clinical translation, the convergence of smart nanomaterials, data-driven models, and sustainable engineering offers a compelling vision for carbon-based diagnostics and therapeutics. Future efforts must prioritize AI-guided hybrid platforms that balance performance, biocompatibility, and ecological impact. Establishing standardized databases and open-source models will boost reproducibility. Ultimately, with AI integration as a defining factor, GCDs are uniquely positioned to transform global health innovation at the intersection of materials science, computational biology, and precision medicine.

Looking further ahead, the role of AI is set to evolve from predictive modeling to proactive creation. Generative AI models, such as generative adversarial networks and transformers, will optimize existing parameters and be capable of the de novo design of entirely novel GCD structures. By providing a set of desired therapeutic criteria—for instance, the ability to cross the blood-brain barrier while targeting a specific receptor with a defined fluorescence wavelength—these models could generate candidate molecular structures computationally. This would fundamentally shift nanomaterial discovery from a reactive, experimental process to a proactive, design-driven paradigm, dramatically accelerating the development of next-generation theranostics.

The convergence of AI with multi omics data paves the way for the concept of digital twins in personalized medicine. In the future, it may be possible to create a high-fidelity computational model of a patient’ s specific pathophysiology based on their genomic, proteomic, and metabolic data. Within this digital twin, the therapeutic efficacy and potential toxicity of a newly designed GCD-based nanocarrier could be simulated in silico before any physical administration. This approach would enable the pre selection of the safest therapy and create a dynamic platform for personalized dose adjustment in response to simulated biological feedback, directly realizing the adaptive therapeutic goals discussed in section 4.2. This would enable the selection and refinement of the most effective and safest therapy for an individual, representing the ultimate realization of precision nanomedicine.

**Figure 13:**
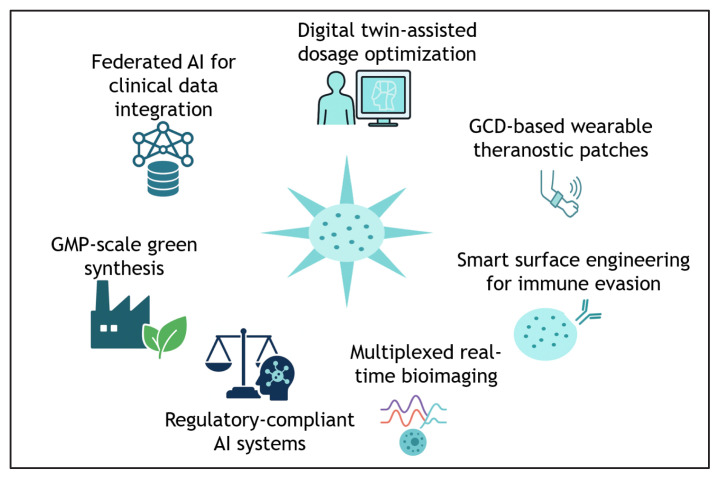
Schematic representation of 7 emerging trends shaping the future of GCD-based theranostics. This figure provides an overview of the key developments that will define the future of the field, from AI-driven solutions for data integration and dosage optimization to advances in manufacturing, bioimaging, and regulatory compliance.

Finally, the principle of green synthesis will expand to encompass the entire lifecycle of GCD-based technologies within a circular economy framework. Future research, guided by AI, will focus not only on sustainable production from biomass but also on designing GCDs for end-of-life biodegradability or recyclability. AI-driven life cycle assessment models could predict the environmental footprint of a nanodevice from its synthesis to its disposal, optimizing for materials that can be safely returned to the biosphere or reclaimed for new applications. This cradle-to-cradle approach will be essential for ensuring that the advances in nanomedicine do not come at an environmental cost, aligning technological innovation with environmental health. Seven major future directions in GCD-based theranostics are conceptually illustrated in Figure 13, highlighting the convergence of AI, regulatory alignment, and sustainable synthesis strategies.

## 9. Conclusions

GCDs show immense promise across drug delivery, diagnostics, and bioimaging due to their tunable photoluminescence, high biocompatibility, and minimal toxicity. Their optical versatility enables multicolor bioimaging and sensitive ratiometric sensing, while their favorable biodistribution and surface functionalization enhance targeted delivery, positioning them as an environmentally conscious option for advanced biomedicine. The integration of AI and ML is a pivotal force in advancing GCDs, transforming their design from iterative experimentation to data-driven, predictive workflows. This AI-driven approach models complex relationships between synthesis conditions and desired properties, accelerating research, reducing resource consumption, and streamlining the discovery of novel GCD compounds with enhanced QY s and specificity for diverse biomedical applications (Shang et al., 2024; Etefa and Dejene, 2025). The influence of AI extends to downstream biomedical applications. AI-guided models are crucial for interpreting complex biological signals from GCD-based sensors. For example, CNN-based computer vision frameworks enable unbiased detection of subcellular features from GCD-enabled images, facilitating early diagnosis. Reinforcement learning systems are proposed for adaptive drug delivery, using GCDs as real-time sensors to modulate drug release based on environmental cues, exemplifying feedback-enabled theranostic systems for precision medicine (Shang et al., 2024). Furthermore, AI automates synthesis optimization, addressing critical bottlenecks like reproducibility and batch-to-batch consistency. This convergence of AI, automation, and green nanotechnology, coupled with the ability of AI to integrate GCD design with multi omics and patient-specific data, paves the way for personalized GCDs in disease detection, monitoring, and treatment.

Despite this promise, widespread commercialization faces significant hurdles, including ensuring long-term toxicity and biodegradability, standardizing synthesis protocols, and developing robust, generalizable AI models for noisy biological data. These challenges necessitate rigorous model validation, standardized datasets, and interpretable AI.

From a sustainability perspective, AI-guided precursor selection and reaction optimization enhance the green credentials of GCDs by minimizing energy and maximizing atom economy (Bajwa et al., 2021; Alowais et al., 2023). This synergy embeds environmental consciousness, scalability, and clinical relevance into nanobiotechnology. Ultimately, AI-enhanced GCDs are poised to profoundly impact global health and environmental sustainability. Their intrinsic benefits, combined with the power of AI to optimize design and analyze complex data, can lead to more accurate disease detection, customized drug delivery, and sophisticated theranostic agents, improving patient outcomes even in resource-limited settings (Javaid et al., 2024; Zuhair et al., 2024). This paradigm strongly aligns with the United Nations sustainable development goals (SDGs), particularly SDG 3 (Good Health and Well being), SDG 9 (Industry, Innovation, and Infrastructure), and SDG 12 (Responsible Consumption and Production) for resilient, climate-smart, and ethically aligned healthcare ecosystems. The future of GCD innovation demands a deeply interdisciplinary approach to overcome current scientific and technical challenges.

## Figures and Tables

**Table:** Comparative analysis of top-down vs bottom- up synthesis of GCDs.

Parameter	Top-down approaches	Bottom-up approaches
**Yield**	**Low to moderate** (e.g., laser ablation: 4–10%). Electrochemical and chemical oxidation methods may reach higher yields but often require harsh conditions and yield losses during purification.	**Typically high** (e.g., hydrothermal and microwave synthesis often achieve 20–70% or more), especially with organic precursors or biomass.
**Scalability**	**Limited** scalability due to batch processes and equipment constraints (e.g., arc discharge and laser ablation). Electrochemical methods require electrode uniformity.	**High** scalability, especially for hydrothermal and microwave methods. Compatible with continuous flow systems and simple upscaling.
**Cost**	**Higher** equipment and energy costs (e.g., high-power lasers, electrochemical setups). Extensive purification increases post synthesis costs.	**Lower** overall cost due to low-cost precursors (e.g., biomass), simpler equipment, and minimal purification requirements.
**Purification demand**	Often requires multiple purification steps due to byproducts and metal residues , which impacts material recovery and sustainability.	Generally fewer purification steps, especially in green methods using mild solvents and low-toxicity reagents.
**Sustainability**	**Less** environmentally friendly due to strong oxidants and high energy input. Greener variants are under development.	**More** sustainable, especially when using biomass and water-based solvents under mild reaction conditions.
**References**	Chai et al. (2022) ; Desmond et al. (2021); Jing et al. (2023); Rocco et al. (2023); Ullal et al. (2022); Zhang and Wada, (2021).	Founti et al. (2024); Kayani et al. (2024); Ahmad et al. (2025); Huang and Ren (2025); Sahana et al. (2023); Singh et al. (2025); Wang et al. (2025).

## Footnotes

1 Agarwal A, Goenka GD, Chumbhale N (2025). Quantum Dots in Neuroimaging: Machine Learning for Brainwave Analysis and Toxicity Assessment [online]. Website: https://ssrn.com/abstract=5237091 [accessed 01 10 2025].

2 Agarwal A, Chumbhale N (2025). Quantum Dots in Neuroimaging: Machine Learning for Brainwave Analysis and Toxicity Assessment [online]. Website: https://ssrn.com/abstract=5237091 [accessed 01 10 2025].

3 European Commission (2024). Artificial Intelligence Act Proposal: Regulation of high-risk AI systems in medical devices [online]. Website https://digital-strategy.ec.europa.eu/en/policies/regulatory-framework-ai [accessed 01 10 2025].

4 WIPO (2023). World Intellectual Property Indicators 2023 [online]. Website: https://www.wipo.int/publications/en/details.jsp?id=4644 [accessed 01 10 2025].

5 Allied Market Research (2024). Carbon Quantum Dots Market by Application and End Use: Global Opportunity Analysis and Industry Forecast, 2024–2033 [online]. Website: https://www.alliedmarketresearch.com/carbon-quantum-dots-market [accessed 01 10 2025].

6 Market.us (2024). AI in Nanotechnology Market Size, Growth Trends and Forecast 2023 to 2032 [online]. Website: https://market.us/report/ai-in-nanotechnology-market/ [accessed 01 10 2025].

7 Grand View Research (2024). Nanotechnology Market Size, Share & Trends Analysis Report By Type (Nanodevices, Nanosensors), By Application, By Region, And Segment Forecasts, 2023–2030 [online]. Website: https://www.grandviewresearch.com/industry-analysis/nanotechnology-market [accessed 01 10 2025].

8 Fortune Business Insights (2024). Carbon Nanomaterials Market Size, Share & COVID-19 Impact Analysis [online]. Website: https://www.fortunebusinessinsights.com/carbon-nanomaterials-market-104591 [accessed 01 10 2025].

## References

[b1-tjb-49-05-498] Abady MM Mohammed DM Soliman TN Shalaby RA Sakr FA 2025 Sustainable synthesis of nanomaterials using different renewable sources Bulletin of the National Research Centre 49 1 1 28 10.1186/S42269-025-01316-4

[b2-tjb-49-05-498] Adil M Menon VG Balasubramanian V Alotaibi SR Song H 2023 Survey: Self-Empowered Wireless Sensor Networks Security Taxonomy, Challenges, and Future Research Directions IEEE Sensors Journal 23 18 20519 20535 10.1109/JSEN.2022.3216824

[b3-tjb-49-05-498] Agboklu MA Adrah F Agbenyo PM Nyavor H 2024 From Bits to Atoms: Machine Learning and Nanotechnology for Cancer Therapy Journal of Nanotechnology Research 06 01 10.26502/jnr.2688-85210042

[b4-tjb-49-05-498] Agrawal A Keçili R Ghorbani-Bidkorbeh F Hussain CM 2021 Green miniaturized technologies in analytical and bioanalytical chemistry TrAC Trends in Analytical Chemistry 143 116383 10.1016/J.TRAC.2021.116383

[b5-tjb-49-05-498] Ahmad S Salma U Alam MZ Mohasin M Parveen H 2025 Introduction to Green Carbon Dots ACS Symposium Series 1 26 10.1021/BK-2025-1506.CH001

[b6-tjb-49-05-498] Ahn J Song Y Kwon JE Woo J Kim H 2019 Characterization of food waste-driven carbon dot focusing on chemical structural, electron relaxation behavior and Fe3+ selective sensing Data in Brief 25 104038 10.1016/J.DIB.2019.104038 31194181 PMC6554359

[b7-tjb-49-05-498] Aksu M Güzdemir Ö 2025 Food Waste-Derived Carbon Quantum Dots and Their Applications in Food Technology: A Critical Review Food and Bioprocess Technology 1 26 10.1007/S11947-025-03854-1

[b8-tjb-49-05-498] Ala M Suriano C Bartoli M Tagliaferro A 2025 An overview on metal-doped carbon dots uses for biomedical applications Materials Today Quantum 5 100030 10.1016/J.MTQUAN.2025.100030

[b9-tjb-49-05-498] Alagumalai A Shou W Mahian O Aghbashlo M Tabatabaei M 2022 Self-powered sensing systems with learning capability Joule 6 7 1475 1500 10.1016/j.joule.2022.06.001

[b10-tjb-49-05-498] Alowais SA Alghamdi SS Alsuhebany N Alqahtani T Alshaya AI 2023 Revolutionizing healthcare: the role of artificial intelligence in clinical practice BMC Medical Education 23 1 1 15 10.1186/S12909-023-04698-Z 37740191 PMC10517477

[b11-tjb-49-05-498] Amin M Huang W Seynhaeve ALB Ten Hagen TLM 2020 Hyperthermia and Temperature-Sensitive Nanomaterials for Spatiotemporal Drug Delivery to Solid Tumors Pharmaceutics 12 11 1007 10.3390/PHARMACEUTICS12111007 33105816 PMC7690578

[b12-tjb-49-05-498] Anaam A Al-antari MA Gofuku A 2023 A deep learning self-attention cross residual network with Info-WGANGP for mitotic cell identification in HEp-2 medical microscopic images Biomedical Signal Processing and Control 86 105191 10.1016/j.bspc.2023.105191

[b13-tjb-49-05-498] Anastas P Eghbali N 2009 Green Chemistry: Principles and Practice Chemical Society Reviews 39 1 301 312 10.1039/B918763B 20023854

[b14-tjb-49-05-498] Andresen TL Thompson DH Kaasgaard T 2010 Enzyme-triggered nanomedicine: Drug release strategies in cancer therapy (Invited Review) Molecular Membrane Biology 27 7 353 10.3109/09687688.2010.515950 20939771 PMC6889806

[b15-tjb-49-05-498] Anpalagan K Yin H Cole I Zhang T Lai DTH 2024 Quantum Yield Enhancement of Carbon Quantum Dots Using Chemical-Free Precursors for Sensing Cr (VI) Ions Inorganics 12 4 96 10.3390/INORGANICS12040096

[b16-tjb-49-05-498] Arble C Jia M Newberg JT 2018 Lab-based ambient pressure X-ray photoelectron spectroscopy from past to present Surface Science Reports 73 2 37 57 10.1016/J.SURFREP.2018.02.002

[b17-tjb-49-05-498] Ateia EE Rabie O Mohamed AT 2024 Assessment of the correlation between optical properties and CQD preparation approaches The European Physical Journal Plus 139 1 1 12 10.1140/EPJP/S13360-023-04811-7

[b18-tjb-49-05-498] Ayaz F Alas MO Genc R 2020 Differential Immunomodulatory Effect of Carbon Dots Influenced by the Type of Surface Passivation Agent Inflammation 43 2 777 783 10.1007/s10753-019-01165-0 31873835

[b19-tjb-49-05-498] Bajwa J Munir U Nori A Williams B 2021 Artificial intelligence in healthcare: transforming the practice of medicine Future Healthcare Journal 8 2 e188 10.7861/FHJ.2021-0095 34286183 PMC8285156

[b20-tjb-49-05-498] Bao X Yuan Y Chen J Zhang B Li D Zhou D Jing P Xu G Wang Y Hola K Shen D Wu C Song L Liu C Sboril R Qu S 2018 In vivo theranostics with near-infrared-emitting carbon dots—highly efficient photothermal therapy based on passive targeting after intravenous administration Light: Science & Applications 7 1 91 10.1038/s41377-018-0090-1 PMC6249234 30479757

[b21-tjb-49-05-498] Bavya V Rajan TPD Suresh KI 2025 Design of Fluorescence Enhancing Sensor for Mercury Detection via Bamboo Cellulose-Derived Carbon Dots Langmuir 10.1021/acs.langmuir.4c03942 39791473

[b22-tjb-49-05-498] Bellucci S Speranza G 2022 Characterization of Carbon Nanostructures by Photoelectron Spectroscopies Materials 15 13 4434 10.3390/MA15134434 35806559 PMC9267296

[b23-tjb-49-05-498] Bennett JA Abolhasani M 2022 Autonomous chemical science and engineering enabled by self-driving laboratories Current Opinion in Chemical Engineering 36 100831 10.1016/j.coche.2022.100831

[b24-tjb-49-05-498] Betz UAK Arora L Assal RA Azevedo H Baldwin J 2023 Game changers in science and technology - now and beyond Technological Forecasting and Social Change, 193 122588 10.1016/j.techfore.2023.122588

[b25-tjb-49-05-498] Bhavikatti SK Zainuddin SLA Ramli RB Nadaf SJ Dandge PB 2024 Green-synthesized carbon dots from ginger: Multifunctional agents against oral pathogens with biocompatibility in human gingival fibroblast cells Current Plant Biology 40 100392 10.1016/J.CPB.2024.100392

[b26-tjb-49-05-498] Bodele VG Lade SN Undirwade DS Umekar MJ Burle SS 2025 Transforming oncology with carbon quantum dots: Synthesis, properties, and therapeutic potential Next Nanotechnology 7 100181 10.1016/J.NXNANO.2025.100181

[b27-tjb-49-05-498] Botifoll M Pinto-Huguet I Arbiol J 2022 Machine learning in electron microscopy for advanced nanocharacterization: current developments, available tools and future outlook Nanoscale horizons, 7 12 1427 1477 10.1039/D2NH00377E 36239693

[b28-tjb-49-05-498] Bradford G Lopez J Ruza J Stolberg MA Osterude R 2023 Chemistry-informed machine learning for polymer electrolyte discovery ACS Central Science 9 2 206 216 36844492 10.1021/acscentsci.2c01123 PMC9951296

[b29-tjb-49-05-498] Butler KT Davies DW Cartwright H Isayev O Walsh A 2018 Machine learning for molecular and materials science Nature 559 7715 547 555 30046072 10.1038/s41586-018-0337-2

[b30-tjb-49-05-498] Carini C Seyhan AA 2024 Tribulations and future opportunities for artificial intelligence in precision medicine Journal of Translational Medicine 22 411 10.1186/s12967-024-05067-0 38702711 PMC11069149

[b31-tjb-49-05-498] Cave J Christiono A Schiavone C Pownall HJ Cristini V 2025 ) Rational Design of Safer Inorganic Nanoparticles via Mechanistic Modeling-Informed Machine Learning ACS Nano 10.1021/acsnano.5c03590 PMC12177941 40460056

[b32-tjb-49-05-498] Chahardehi AM Arsad H Lim V 2020 Zebrafish as a Successful Animal Model for Screening Toxicity of Medicinal Plants Plants 9 10 1345 10.3390/PLANTS9101345 33053800 PMC7601530

[b33-tjb-49-05-498] Chai Y Feng Y Zhang K Li J Vasile S 2022 Preparation of Fluorescent Carbon Dots Composites and Their Potential Applications in Biomedicine and Drug Delivery—A Review Pharmaceutics 14 11 2482 10.3390/PHARMACEUTICS14112482 36432673 PMC9697445

[b34-tjb-49-05-498] Chakroborty S Nath N Sahoo S Singh BP Bal T 2025 A review of emerging trends in nanomaterial-driven AI for biomedical applications Nanoscale Advances 7 12 3619 3630 10.1039/d5na00032g 40370571 PMC12071765

[b35-tjb-49-05-498] Chávez-Angel E Eriksen MB Castro-Alvarez A Garcia JH Botifoll M 2025 Applied Artificial Intelligence in Materials Science and Material Design Advanced Intelligent Systems 2400986.

[b36-tjb-49-05-498] Chen W Liu X Zhang S Chen S 2023 Artificial intelligence for drug discovery: Resources, methods, and applications Molecular Therapy Nucleic Acids 31 691 702 36923950 10.1016/j.omtn.2023.02.019 PMC10009646

[b37-tjb-49-05-498] Chen Z Li Z He H Liu J Deng J 2024 Ratiometric fluorescence sensor based on deep learning for rapid and user-friendly detection of tetracycline antibiotics Food Chemistry 450 10.1016/j.foodchem.2024.138961 38640544

[b38-tjb-49-05-498] Chen Z Liao T Wan L Kuang Y Liu C 2021 Dual-stimuli responsive near-infrared emissive carbon dots/hollow mesoporous silica-based integrated theranostics platform for real-time visualized drug delivery Nano Research 14 11 4264 4273 10.1007/s12274-021-3624-4

[b39-tjb-49-05-498] Choudhary K DeCost B Chen C Jain A Tavazza F 2022 Recent advances and applications of deep learning methods in materials science npj Computational Materials 8 1 59

[b40-tjb-49-05-498] Chung CY Chen YJ Kang CH Lin HY Huang CC 2021 Toxic or not toxic, that is the carbon quantum dot’s question: A comprehensive evaluation with zebrafish embryo, eleutheroembryo, and adult models Polymers 13 10 1598 34063447 10.3390/polym13101598 PMC8155906

[b41-tjb-49-05-498] Ciftci F Özarslan AC Kantarci İC Yelkenci A Tavukcuoglu O 2025 Advances in Drug Targeting, Drug Delivery, and Nanotechnology Applications: Therapeutic Significance in Cancer Treatment Pharmaceutics 17 1 121 10.3390/PHARMACEUTICS17010121 39861768 PMC11769154

[b42-tjb-49-05-498] Cui D Zhai S Yang Y Wu Y Li J 2022 A Label-Free Electrochemical Impedance Genosensor Coupled with Recombinase Polymerase Amplification for Genetically Modified Maize Detection Agriculture (Switzerland) 12 4 10.3390/agriculture12040454

[b43-tjb-49-05-498] Cui L Ren X Sun M Liu H Xia L 2021 Carbon Dots: Synthesis, Properties and Applications Nanomaterials 11 12 3419 10.3390/NANO11123419 34947768 PMC8705349

[b44-tjb-49-05-498] Dada SN Babanyinah GK Tetteh MT Palau VE Walls ZF 2022 Covalent and Noncovalent Loading of Doxorubicin by Folic Acid-Carbon Dot Nanoparticles for Cancer Theranostics ACS Omega 7 27 23322 23331 35847251 10.1021/acsomega.2c01482 PMC9280931

[b45-tjb-49-05-498] Dai D Zhang C Thuy NTD Zhao G Lu W 2022 Strong fluorescence quenching of carbon dots by mercury(II) ions: Ground-state electron transfer and diminished oscillator strength Diamond and Related Materials 126 109076 10.1016/J.DIAMOND.2022.109076

[b46-tjb-49-05-498] Das S Mondal S Ghosh D 2023 Carbon quantum dots in bioimaging and biomedicines Frontiers in Bioengineering and Biotechnology 11 1333752 38318419 10.3389/fbioe.2023.1333752 PMC10841552

[b47-tjb-49-05-498] Das S Ngashangva L Goswami P 2021 Carbon Dots: An Emerging Smart Material for Analytical Applications Micromachines 12 1 84 10.3390/MI12010084 33467583 PMC7829846

[b48-tjb-49-05-498] de Curtò J de Zarzà I Roig G Calafate CT 2024 Large Language Model-Informed X-ray Photoelectron Spectroscopy Data Analysis Signals 5 2 181 201 10.3390/signals5020010

[b49-tjb-49-05-498] de la Rica R Aili D Stevens MM 2012 Enzyme-responsive nanoparticles for drug release and diagnostics Advanced Drug Delivery Reviews 64 11 967 978 10.1016/J.ADDR.2012.01.002 22266127

[b50-tjb-49-05-498] del Giudice G Serra A Pavel A Torres Maia M Saarimäki LA 2024 A Network Toxicology Approach for Mechanistic Modelling of Nanomaterial Hazard and Adverse Outcomes Advanced Science 11 32 10.1002/advs.202400389 PMC11348149 38923832

[b51-tjb-49-05-498] Desmond LJ Phan AN Gentile P 2021 Critical overview on the green synthesis of carbon quantum dots and their application for cancer therapy Environmental Science: Nano 8 4 848 862 10.1039/D1EN00017A

[b52-tjb-49-05-498] Dhumal P Chakraborty S Ibrahim B Kaur M Valsami-Jones E 2024 Green-synthesised carbon nanodots: A SWOT analysis for their safe and sustainable innovation Journal of Cleaner Production 480 144115 10.1016/J.JCLEPRO.2024.144115

[b53-tjb-49-05-498] Dias C Vasimalai N Sárria MP Pinheiro I Vilas-Boas V 2019 Biocompatibility and Bioimaging Potential of Fruit- Based Carbon Dots Nanomaterials 9 2 199 10.3390/NANO9020199 30717497 PMC6409625

[b54-tjb-49-05-498] Díaz-García D Díaz-Sánchez M Álvarez-Conde J Gómez-Ruiz S 2024 Emergence of Quantum Dots as Innovative Tools for Early Diagnosis and Advanced Treatment of Breast Cancer ChemMedChem 19 16 e202400172 10.1002/CMDC.202400172 38724442 PMC13494823

[b55-tjb-49-05-498] Ding H Yu SB Wei JS Xiong HM 2016 Full-color light-emitting carbon dots with a surface-state-controlled luminescence mechanism ACS Nano 10 1 484 491 doi: 10.1021/acsnano.5b05406 26646584

[b56-tjb-49-05-498] Dokuzparmak E Sezer E Güner T Yaşar E Özçelïk H 2025 Hybrid Intelligence-Driven Nanopolymeric Sensor for Precise Electrochemical Vitamin C Analysis, Free from LoD: Application in Real Lemon Juice ACS Applied Electronic Materials 7 15 6980 6993

[b57-tjb-49-05-498] Dong Z Qi J Yue L Zhou H Chen L 2024 Biomass-based carbon quantum dots and their agricultural applications Plant Stress 11 100411 10.1016/J.STRESS.2024.100411

[b58-tjb-49-05-498] Döring A Rogach AL 2022 Utilizing Deep Learning to Enhance Optical Sensing of Ethanol Content Based on Luminescent Carbon Dots ACS Applied Nano Materials 5 8 11208 11218 10.1021/acsanm.2c02351

[b59-tjb-49-05-498] Dugam S Nangare S Patil P Jadhav N 2021 Carbon dots: A novel trend in pharmaceutical applications Annales Pharmaceutiques Françaises 79 4 335 345 10.1016/J.PHARMA.2020.12.002 33383021

[b60-tjb-49-05-498] Duman AN Jalilov AS 2024 Machine learning for carbon dot synthesis and applications Materials Advances 10.1039/d4ma00505h

[b61-tjb-49-05-498] Elugoke SE Uwaya GE Quadri TW Ebenso EE 2024 Carbon Quantum Dots: Basics, Properties, and Fundamentals ACS Symposium Series 1465 3 42 10.1021/bk-2024-1465.ch001

[b62-tjb-49-05-498] Enyoh CE Wang Q Wang W Suzuki M Masuda G 2025 Green One-Step Synthesis and Characterization of Fluorescent Carbon Quantum Dots from PET Waste as a Dual-Mode Sensing Probe for Pd(II), Ciprofloxacin, and Fluoxetine via Fluorescence Quenching and Enhancement Mechanisms Surfaces 8 2 24 10.3390/SURFACES8020024

[b63-tjb-49-05-498] Esmaeili Y Toiserkani F Qazanfarzadeh Z Ghasemlou M Naebe M 2025 Unlocking the potential of green-engineered carbon quantum dots for sustainable packaging biomedical applications and water purification Advances in Colloid and Interface Science 338 103414 10.1016/J.CIS.2025.103414 39889506

[b64-tjb-49-05-498] Etefa HF Dejene FB 2025 Applications of Green Carbon Dots in Personalized Diagnostics for Precision Medicine International Journal of Molecular Sciences 26 7 10.3390/ijms26072846 PMC11988419 40243410

[b65-tjb-49-05-498] Farshbaf M Davaran S Rahimi F Annabi N Salehi R 2018 Carbon quantum dots: recent progresses on synthesis, surface modification and applications Artificial Cells, Nanomedicine and Biotechnology 46 7 1331 1348 10.1080/21691401.2017.1377725 28933188

[b66-tjb-49-05-498] Fatahi Z Esfandiari N Ehtesabi H Bagheri Z Tavana H 2019 Physicochemical and cytotoxicity analysis of green synthesis carbon dots for cell imaging EXCLI Journal 18 454 466 10.17179/EXCLI2019-1465 31423124 PMC6694706

[b67-tjb-49-05-498] Fathi-karkan S Nasiri S Hasannia M Rahdar A Nouri M 2025 Green carbon dots and theranostic applications Journal of Drug Delivery Science and Technology 108 106861 10.1016/J.JDDST.2025.106861

[b68-tjb-49-05-498] Forest V 2022 Experimental and Computational Nanotoxicology—Complementary Approaches for Nanomaterial Hazard Assessment Nanomaterials 12 8 1346 10.3390/nano12081346 35458054 PMC9031966

[b69-tjb-49-05-498] Founti M Nikolaidis GN Usman M Cheng S 2024 Recent Trends and Advancements in Green Synthesis of Biomass-Derived Carbon Dots Eng 5 3 2223 2263 10.3390/ENG5030116

[b70-tjb-49-05-498] Gao L Lin J Wang L Du L 2024a Machine Learning-Assisted Design of Advanced Polymeric Materials Accounts of Materials Research 5 5 571 584 10.1021/accountsmr.3c00288

[b71-tjb-49-05-498] Gao XJ Ciura K Ma Y Mikolajczyk A Jagiello K 2024b Toward the Integration of Machine Learning and Molecular Modeling for Designing Drug Delivery Nanocarriers Advanced Materials 10.1002/adma.202407793 39252670

[b72-tjb-49-05-498] Garcia GR Noyes PD Tanguay RL 2016 Advancements in zebrafish applications for 21st century toxicology Pharmacology & Therapeutics 161 11 10.1016/J.PHARMTHERA.2016.03.009 27016469 PMC4851906

[b73-tjb-49-05-498] Ge W De Silva R Fan Y Sisson SA Stenzel MH 2025 Machine learning in polymer research Advanced Materials 37 11 2413695 39924835 10.1002/adma.202413695 PMC11923530

[b74-tjb-49-05-498] Ghataty DS Amer RI Amer MA Abdel Rahman MF Shamma RN 2023 Green Synthesis of Highly Fluorescent Carbon Dots from Bovine Serum Albumin for Linezolid Drug Delivery as Potential Wound Healing Biomaterial: Bio-Synergistic Approach, Antibacterial Activity, and In Vitro and Ex Vivo Evaluation Pharmaceutics 15 1 234 36678866 10.3390/pharmaceutics15010234 PMC9862409

[b75-tjb-49-05-498] Gholap AD Uddin MJ Faiyazuddin M Omri A Gowri S 2024 Advances in artificial intelligence for drug delivery and development: A comprehensive review Computers in Biology and Medicine 178 108702 10.1016/j.compbiomed.2024.108702 38878397

[b76-tjb-49-05-498] Ghosh S Barg S Jeong SM Ostrikov K 2020 Heteroatom-Doped and Oxygen-Functionalized Nanocarbons for High- Performance Supercapacitors Advanced Energy Materials 10 32 2001239 10.1002/AENM.202001239;PAGE:STRING:ARTICLE/CHAPTER

[b77-tjb-49-05-498] Goularte dos Santos K Gracher Riella H Soares C Padoin N 2025 OptiDots_v1.0: A Comprehensive Database for Machine Learning-Driven Optimization of Green Synthesis Carbon Dots 10.26434/chemrxiv-2025-2p16l

[b78-tjb-49-05-498] Goumas G Vlachothanasi EN Fradelos EC Mouliou DS 2025 Biosensors, artificial intelligence biosensors, false results and novel future perspectives Diagnostics 15 8 1037 40310427 10.3390/diagnostics15081037 PMC12025796

[b79-tjb-49-05-498] Greener JG Kandathil SM Moffat L Jones DT 2022 A guide to machine learning for biologists Nature Reviews Molecular Cell Biology 23 1 40 55 10.1038/s41580-021-00407-0 34518686

[b80-tjb-49-05-498] Grüll H Langereis S 2012 Hyperthermia-triggered drug delivery from temperature-sensitive liposomes using MRI-guided high intensity focused ultrasound Journal of Controlled Release 161 2 317 327 10.1016/J.JCONREL.2012.04.041 22565055

[b81-tjb-49-05-498] Guerrini L Alvarez-Puebla RA Pazos-Perez N 2018 Surface Modifications of Nanoparticles for Stability in Biological Fluids Materials 11 7 1154 10.3390/MA11071154 29986436 PMC6073273

[b82-tjb-49-05-498] Guo H Lu Y Lei Z Bao H Zhang M 2024 Machine learning-guided realization of full-color high-quantum-yield carbon quantum dots Nature Communications 15 1 10.1038/s41467-024-49172-6 PMC11156924 38844440

[b83-tjb-49-05-498] Guo S Popp J Bocklitz T 2021 Chemometric analysis in Raman spectroscopy from experimental design to machine learning–based modeling Nature Protocols 16 5426 5459 10.1038/s41596-021-00620-3 34741152

[b84-tjb-49-05-498] Han Y Tang B Wang L Bao H Lu Y 2020 Machine-learning-driven synthesis of carbon dots with enhanced quantum yields ACS Nano 14 11 14761 14768 32960048 10.1021/acsnano.0c01899

[b85-tjb-49-05-498] Hasan N Muthu M Hakami O Gopal J 2025 Assessing the Sustainability of Energy-Related Nanomaterial Synthesis: Emphasizing the Need for Energy- Efficient Nanomaterial Preparation Techniques Energies 18 3 523 10.3390/EN18030523

[b86-tjb-49-05-498] Hatimuria M Phukan P Bag S Ghosh J Gavvala K 2023 Green Carbon Dots: Applications in Development of Electrochemical Sensors, Assessment of Toxicity as Well as Anticancer Properties Catalysts 13 3 537 10.3390/CATAL13030537

[b87-tjb-49-05-498] Havrdova M Hola K Skopalik J Tomankova K Petr M 2016 Toxicity of carbon dots – Effect of surface functionalization on the cell viability, reactive oxygen species generation and cell cycle Carbon 99 238 248 10.1016/J.CARBON.2015.12.027

[b88-tjb-49-05-498] He S Barón A Munteanu CR de Bilbao B Casañola-Martin GM 2025 Drug Release Nanoparticle System Design: Data Set Compilation and Machine Learning Modeling ACS Applied Materials and Interfaces 10.1021/acsami.4c16800 39800937

[b89-tjb-49-05-498] Herdiana Y Febrina E Nurhasanah S Gozali D Elamin KM 2024 Drug Loading in Chitosan- Based Nanoparticles Pharmaceutics 16 8 1043 10.3390/PHARMACEUTICS16081043 39204388 PMC11359066

[b90-tjb-49-05-498] Hosni Z Achour S Saadi F Chen Y Al Qaraghuli M 2025 Machine learning-driven nanoparticle toxicity Ecotoxicology and Environmental Safety 299 118340 10.1016/j.ecoenv.2025.118340 40393320

[b91-tjb-49-05-498] Hu Q Katti PS Gu Z 2014 Enzyme-Responsive Nanomaterials for Controlled Drug Delivery Nanoscale 6 21 12273 12290 10.1039/C4NR04249B 25251024 PMC4425417

[b92-tjb-49-05-498] Huang Z Ren L 2025 Large Scale Synthesis of Carbon Dots and Their Applications: A Review Molecules 30 4 774 40005085 10.3390/molecules30040774 PMC11857885

[b93-tjb-49-05-498] Hulme J 2022 Application of Nanomaterials in the Prevention, Detection, and Treatment of Methicillin-Resistant Staphylococcus aureus (MRSA Pharmaceutics 14 4 805 10.3390/pharmaceutics14040805 35456638 PMC9030647

[b94-tjb-49-05-498] Huo Z Yu Z Xu W Xu S 2024 ) Super-Resolution Microscopic Imaging of Lipid Droplets in Living Cells via Carbonized Polymer Dot-Based Polarity-Responsive Nanoprobe ACS Measurement Science Au 10.1021/acsmeasuresciau.4c00049 PMC11487779 39430970

[b95-tjb-49-05-498] Hussain MM Khan WU Ahmed F Wei Y Xiong H 2023 Recent developments of Red/NIR carbon dots in biosensing, bioimaging, and tumor theranostics Chemical Engineering Journal 465 143010 10.1016/j.cej.2023.143010

[b96-tjb-49-05-498] Javaid M Haleem A Haleem Khan I Singh RP Ali Khan A 2024 Industry 4.0 and circular economy for bolstering healthcare sector: A comprehensive view on challenges, implementation, and futuristic aspects Biomedical Analysis 1 2 174 198 10.1016/J.BIOANA.2024.06.001

[b97-tjb-49-05-498] Ji J Zhou J Yang Z Lin Q Coello CAC 2023 AutoDock Koto: A Gradient Boosting Differential Evolution for Molecular Docking IEEE Transactions on Evolutionary Computation 27 6 1648 1662 10.1109/TEVC.2022.3225632

[b98-tjb-49-05-498] Ji Z Guo W Wood EL Liu J Sakkiah S 2022 Machine Learning Models for Predicting Cytotoxicity of Nanomaterials Chemical Research in Toxicology 35 2 125 139 10.1021/acs.chemrestox.1c00310 35029374

[b99-tjb-49-05-498] Jia X Wang T Zhu H 2023 Advancing Computational Toxicology by Interpretable Machine Learning Environmental Science and Technology 57 46 17690 17706 10.1021/acs.est.3c00653 37224004 PMC10666545

[b100-tjb-49-05-498] Jiang J Cui X Huang Y Yan D Wang B 2024 Advances and Prospects in Integrated Nano-oncology Nano Biomedicine and Engineering 16 2 152 187 10.26599/NBE.2024.9290060

[b101-tjb-49-05-498] Jiang K Feng X Gao X Wang Y Cai C 2019 Preparation of Multicolor Photoluminescent Carbon Dots by Tuning Surface States Nanomaterials 9 4 529 10.3390/NANO9040529 30987120 PMC6523770

[b102-tjb-49-05-498] Jin K Wang W Qi G Peng X Gao H 2023 An explainable machine-learning approach for revealing the complex synthesis path-property relationships of nanomaterials Nanoscale 15 37 15358 15367 10.1039/d3nr02273k 37698588

[b103-tjb-49-05-498] Jin X Liu C Xu T Su L Zhang X 2020 Artificial intelligence biosensors: Challenges and prospects Biosensors and Bioelectronics 165 112412 32729531 10.1016/j.bios.2020.112412

[b104-tjb-49-05-498] Jing HH Bardakci F Akgöl S Kusat K Adnan M 2023 Green Carbon Dots: Synthesis, Characterization, Properties and Biomedical Applications Journal of Functional Biomaterials 14 1 27 10.3390/JFB14010027 36662074 PMC9863160

[b105-tjb-49-05-498] Jing HH Shati AA Alfaifi MY Elbehairi SEI Sasidharan S 2025 The future of plant based green carbon dots as cancer Nanomedicine: From current progress to future Perspectives and beyond Journal of Advanced Research 67 133 159 10.1016/J.JARE.2024.01.034 38320729 PMC11725112

[b106-tjb-49-05-498] Jorns M Pappas D Tagmatarchis N Kelarakis A 2021 A Review of Fluorescent Carbon Dots, Their Synthesis, Physical and Chemical Characteristics, and Applications Nanomaterials 11 6 1448 10.3390/NANO11061448 34070762 PMC8228846

[b107-tjb-49-05-498] Junaid MAL 2025 Artificial intelligence driven innovations in biochemistry A review of emerging research frontiers Biomolecules and Biomedicine 25 4 739 39819459 10.17305/bb.2024.11537 PMC11959397

[b108-tjb-49-05-498] Junyaprasert VB Thummarati P 2023 Innovative Design of Targeted Nanoparticles: Polymer–Drug Conjugates for Enhanced Cancer Therapy Pharmaceutics 15 9 2216 10.3390/PHARMACEUTICS15092216 37765185 PMC10537251

[b109-tjb-49-05-498] Kar DK VP Si S Panigrahi H Mishra S 2024 Carbon dots and their polymeric nanocomposites: insight into their synthesis, photoluminescence mechanisms, and recent trends in sensing applications ACS Omega 9 10 11050 11080 38497004 10.1021/acsomega.3c07612 PMC10938319

[b110-tjb-49-05-498] Kang SH Kwon JY Lee JK Seo YR 2013 Recent Advances in In Vivo Genotoxicity Testing: Prediction of Carcinogenic Potential Using Comet and Micronucleus Assay in Animal Models Journal of Cancer Prevention 18 4 277 10.15430/JCP.2013.18.4.277 25337557 PMC4189446

[b111-tjb-49-05-498] Kang Z Wang Y Song H Wang X Zhang YHPJ 2024 A wearable and flexible lactic-acid/O2 biofuel cell with an enhanced air-breathing biocathode Biosensors and Bioelectronic, 246 115845 10.1016/j.bios.2023.115845 38008057

[b112-tjb-49-05-498] Kannouma RE Kamal AH Hammad MA Mansour FR 2024 Tips and Tricks for Applying luminescent carbon dots in chemical Analysis: Recent Advancements, Obstacles, and future Outlook Microchemical Journal 207 111667 10.1016/j.microc.2024.111667

[b113-tjb-49-05-498] Kanwal A Bibi N Hyder S Muhammad A Ren H 2022 Recent advances in green carbon dots 2015–2022 synthesis, metal ion sensing, and biological applications Beilstein Journal of Nanotechnology 13 1 1068 1107 10.3762/BJNANO.13.93 36262178 PMC9551278

[b114-tjb-49-05-498] Kavgacı M Kalmış HV Eskalen H 2023 Synthesis of fluorescent carbon quantum dots with hydrothermal and solvothermal method application for anticounterfeiting and encryption International Journal of Innovative Engineering Applications 7 1 10.46460/ijiea.1182009

[b115-tjb-49-05-498] Kayani KF Ghafoor D Mohammed SJ Shatery OBA 2024 Carbon dots: synthesis, sensing mechanisms, and potential applications as promising materials for glucose sensors Nanoscale Advances 7 1 42 59 10.1039/D4NA00763H 39583130 PMC11583430

[b116-tjb-49-05-498] Kaymaz SV Nobar HM Sarıgül H Soylukan C Akyüz L 2023 Nanomaterial surface modification toolkit: Principles, components, recipes, and applications Advances in Colloid and Interface Science 322 103035 10.1016/J.CIS.2023.103035 37931382

[b117-tjb-49-05-498] Khan MF Kundu D Gogoi M Shrestha AK Karanth NG 2020 Enzyme-Responsive and Enzyme Immobilized Nanoplatforms for Therapeutic Delivery: An Overview of Research Innovations and Biomedical Applications 165 200 10.1007/978-3-030-47120-0_6

[b118-tjb-49-05-498] Kleinstreuer N Hartung T 2024 Artificial intelligence (AI)—it’s the end of the tox as we know it (and I feel fine)* Archives of Toxicology 98 3 735 754 10.1007/s00204-023-03666-2 38244040 PMC10861653

[b119-tjb-49-05-498] Klojdová I Milota T Smetanová J Stathopoulos C 2023 Encapsulation: A Strategy to Deliver Therapeutics and Bioactive Compounds? Pharmaceuticals 16 3 362 10.3390/PH16030362 36986462 PMC10053789

[b120-tjb-49-05-498] Köhler M Ahire ED Savaliya N Makwana KV Salave S 2025 Protein-Bound Nano-Injectable Suspension: Unveiling the Promises and Challenges Applied Nano 6 2 9 10.3390/APPLNANO6020009

[b121-tjb-49-05-498] Kong J Wei Y Zhou F Shi L Zhao S 2024 Carbon Quantum Dots: Properties, Preparation, and Applications Molecules 29 9 2002 10.3390/MOLECULES29092002 PMC11085940 38731492

[b122-tjb-49-05-498] Kothinti RR 2025 Optimizing Personalized Medicine: Investigating the Role of AI-Driven Genomic Analysis in Tailoring Treatment Plans for Patients with Rare Genetic Disorders 10.5281/ZENODO.14937050

[b123-tjb-49-05-498] Kumar M Kulkarni P Liu S Chemuturi N Shah DK 2023 Nanoparticle biodistribution coefficients: A quantitative approach for understanding the tissue distribution of nanoparticles Advanced Drug Delivery Reviews 194 114708 10.1016/J.ADDR.2023.114708 36682420

[b124-tjb-49-05-498] Kurian M Paul A 2021 Recent trends in the use of green sources for carbon dot synthesis–A short review Carbon Trends 3 100032 10.1016/J.CARTRE.2021.100032

[b125-tjb-49-05-498] Kuznetsova V Coogan Á Botov D Gromova Y Ushakova EV 2024 Expanding the Horizons of Machine Learning in Nanomaterials to Chiral Nanostructures Advanced Materials 36 18 10.1002/adma.202308912 PMC11167410 38241607

[b126-tjb-49-05-498] Kyriakides TR Raj A Tseng TH Xiao H Nguyen R 2021 Biocompatibility of nanomaterials and their immunological properties Biomedical Materials 16 4 10.1088/1748-605X/ABE5FA PMC8357854 33578402

[b127-tjb-49-05-498] Li C Huang J Yuan L Xie W Ying Y 2023 Recent progress of emitting long-wavelength carbon dots and their merits for visualization tracking, target delivery and theranostics Theranostics 13 9 3064 3102 10.7150/thno.80579 37284447 PMC10240821

[b128-tjb-49-05-498] Li J Gong X 2022 The Emerging Development of Multicolor Carbon Dots Small 18 51 10.1002/smll.202205099 36328736

[b129-tjb-49-05-498] Li J Wu S Shi X Cao Y Hao H 2025 Machine Learning-Assisted Biomass-Derived Carbon Dots as Fluorescent Sensor Array for Discrimination of Warfarin and Its Metabolites Langmuir 10.1021/acs.langmuir.4c03945 39797801

[b130-tjb-49-05-498] Lim SY Shen W Gao Z 2014 Carbon quantum dots and their applications Chemical Society Reviews 44 1 362 381 10.1039/C4CS00269E 25316556

[b131-tjb-49-05-498] Lin X Xiong M Zhang J He C Ma X 2021 Carbon dots based on natural resources: Synthesis and applications in sensors Microchemical Journal 160 105604 10.1016/J.MICROC.2020.105604

[b132-tjb-49-05-498] Liu X Wang X Zang D Chang Y Su W 2024 pH-responsive oxygen self-sufficient smart nanoplatform for enhanced tumor chemotherapy and photodynamic therapy Journal of Colloid and Interface Science 675 1080 1090 10.1016/j.jcis.2024.07.113 39018635

[b133-tjb-49-05-498] Lu D Tang Y Gao J Chen Y Wang Q 2019 Green anhydrous assembly of carbon dots via solar light irradiation and its multi-modal sensing performance Dyes and Pigments 165 287 293

[b134-tjb-49-05-498] Lu S Montz B Emrick T Jayaraman A 2022 Semi-supervised machine learning workflow for analysis of nanowire morphologies from transmission electron microscopy images Digital Discovery 1 6 816 833

[b135-tjb-49-05-498] Lodha SR Merchant JG Pillai AJ Gore AH Patil PO 2024 Carbon dot-based fluorescent sensors for pharmaceutical detection: Current innovations, challenges, and future prospects Heliyon 10 24 e41020 10.1016/J.HELIYON.2024.E41020 39759361 PMC11697698

[b136-tjb-49-05-498] Lou XT Zhan L Chen BB 2025 Recent Progress of Carbon Dots in Fluorescence Sensing Inorganics 13 8 256

[b137-tjb-49-05-498] Lou Y Hao X Liao L Zhang K Chen S 2021 Recent advances of biomass carbon dots on syntheses, characterization, luminescence mechanism, and sensing applications Nano Select 2 6 1117 1145 10.1002/NANO.202000232

[b138-tjb-49-05-498] Manandhar S Sjöholm E Bobacka J Rosenholm JM Bansal KK 2021 Polymer-Drug Conjugates as Nanotheranostic Agents Journal of Nanotheranostics 2 1 63 81 10.3390/JNT2010005

[b139-tjb-49-05-498] Mansuriya BD Altintas Z 2021 Carbon Dots: Classification, Properties, Synthesis, Characterization, and Applications in Health Care—An Updated Review 2018–2021 Nanomaterials 11 10 2525 10.3390/NANO11102525 34684966 PMC8541690

[b140-tjb-49-05-498] Mao J He H 2024 Deep learning in fluorescence imaging and analysis Journal of Intelligent Medicine 1 1 42 62 10.1002/jim4.17

[b141-tjb-49-05-498] Marques L Costa B Pereira M Silva A Santos J 2024 Advancing Precision Medicine: A Review of Innovative In Silico Approaches for Drug Development, Clinical Pharmacology and Personalized Healthcare Pharmaceutics 16 3 332 10.3390/pharmaceutics16030332 38543226 PMC10975777

[b142-tjb-49-05-498] Martin Watanabe R Hashimoto K Higashisaka K Haga Y 2023 Evidence-Based Prediction of Cellular Toxicity for Amorphous Silica Nanoparticles ACS Nano 17 11 9987 9999 10.1021/acsnano.2c11968 37254442

[b143-tjb-49-05-498] Masud N Rade J Hasib MHH Krishnamurthy A Sarkar A 2024 Machine learning approaches for improving atomic force microscopy instrumentation and data analytics Frontiers in Physics 12 10.3389/fphy.2024.1347648

[b144-tjb-49-05-498] Mathur P Mori M Patel F Upadhyay K Baxi D 2025 ) Curcuminoid-Linked Lemon-Derived Carbon Dots for pH-Triggered Drug Release ACS Omega 10.1021/acsomega.5c01980 PMC12138824 40488046

[b145-tjb-49-05-498] Matotoka M Masoko P Matotoka M Masoko P 2025 In Vitro Cytotoxicity Determination: Avoiding Pitfalls 10.5772/INTECHOPEN.1008312

[b146-tjb-49-05-498] Mehta VN Jha S Singhal RK Kailasa SK 2014 Preparation of multicolor emitting carbon dots for HeLa cell imaging New Journal of Chemistry 38 12 6152 6160 10.1039/C4NJ00840E

[b147-tjb-49-05-498] Merlen A Buijnsters JG Pardanaud C 2017 A Guide to and Review of the Use of Multiwavelength Raman Spectroscopy for Characterizing Defective Aromatic Carbon Solids: from Graphene to Amorphous Carbons Coatings 7 10 153 10.3390/COATINGS7100153

[b148-tjb-49-05-498] Miao H Wang L Zhuo Y Zhou Z Yang X 2016 Label-free fluorimetric detection of CEA using carbon dots derived from tomato juice Biosensors and Bioelectronics 86 83 89 10.1016/J.BIOS.2016.06.043 27336615

[b149-tjb-49-05-498] Mishra A Jatti VS Sefene EM Paliwal S 2023a Explainable Artificial Intelligence (XAI) and Supervised Machine Learning-based Algorithms for Prediction of Surface Roughness of Additively Manufactured Polylactic Acid (PLA) Specimens Applied Mechanics 4 2 668 698 10.3390/applmech4020034

[b150-tjb-49-05-498] Mishra S Das K Chatterjee S Sahoo P Kundu S 2023b Facile and Green Synthesis of Novel Fluorescent Carbon Quantum Dots and Their Silver Heterostructure: An In Vitro Anticancer Activity and Imaging on Colorectal Carcinoma ACS Omega 8 5 4566 10.1021/ACSOMEGA.2C04964 PMC9909815 36777585

[b151-tjb-49-05-498] Mohammadzadeh kakhki R Mohammadpoor M 2024 Machine learning-driven approaches for synthesizing carbon dots and their applications in photoelectrochemical sensors Inorganic Chemistry Communications 159 11 1859 10.1016/j.inoche.2023.111859

[b152-tjb-49-05-498] Mohanaraman SP Chidambaram R 2024 A holistic review on red fluorescent graphene quantum dots, its synthesis, unique properties with emphasis on biomedical applications Heliyon 10 16 e35760 10.1016/J.HELIYON.2024.E35760 39220916 PMC11365325

[b153-tjb-49-05-498] Molla G Bitew M 2024 Revolutionizing Personalized Medicine: Synergy with Multi-Omics Data Generation, Main Hurdles, and Future Perspectives Biomedicines 12 12 10.3390/biomedicines12122750 PMC11673561 39767657

[b154-tjb-49-05-498] Mu P Han Y Wang J 2025 Gram-Scale Green-Emission Carbon Quantum Dots Produced from Wood via the Hydrothermal Synthesis Method for the Detection of Fe (III Applied Sciences 15 4 1958 10.3390/APP15041958

[b155-tjb-49-05-498] Mujahid M Kına EROL Rustam F Villar MG Alvarado ES 2024 Data oversampling and imbalanced datasets: an investigation of performance for machine learning and feature engineering Journal of Big Data 11 1 87

[b156-tjb-49-05-498] Nayanathara U Yang F Zhang C Wang Y Rossi Herling B 2025 Unravelling the endosomal escape of pH-responsive nanoparticles using the split luciferase endosomal escape quantification assay Biomaterials Science 13 5 1335 1346 10.1039/D4BM01433B 39898829

[b157-tjb-49-05-498] Nguyen KG Baragau IA Gromicova R Nicolaev A Thomson SAJ 2022 Investigating the effect of N-doping on carbon quantum dots structure, optical properties and metal ion screening Scientific Reports 12 1 13806 10.1038/S41598-022-16893-X 35970901 PMC9378613

[b158-tjb-49-05-498] Nie H Li M Li Q Liang S Tan Y 2014 Carbon dots with continuously tunable full-color emission and their application in ratiometric pH sensing Chemistry of Materials 26 10 3104 3112 10.1021/CM5003669

[b159-tjb-49-05-498] Nolte L Tomforde S 2025 A Helping Hand: A Survey About AI-Driven Experimental Design for Accelerating Scientific Research Applied Sciences 15 9 10.3390/app15095208

[b160-tjb-49-05-498] Noorain L Nguyen V Kim HW Nguyen LTB 2023 A Machine Learning Approach for PLGA Nanoparticles in Antiviral Drug Delivery Pharmaceutics 15 2 10.3390/pharmaceutics15020495 PMC9966002 36839817

[b161-tjb-49-05-498] Noviandy TR Idroes GM Mohd Fauzi F Idroes R 2024 Application of Ensemble Machine Learning Methods for QSAR Classification of Leukotriene A4 Hydrolase Inhibitors in Drug Discovery Malacca Pharmaceutics 2 2 68 78 10.60084/mp.v2i2.217

[b162-tjb-49-05-498] Novikov AS 2023 Recent Progress in Theoretical Studies and Computer Modeling of Non-Covalent Interactions Crystals 13 2 10.3390/cryst13020361

[b163-tjb-49-05-498] Odugbemi AI Nyirenda C Christoffels A Egieyeh SA 2024 Artificial intelligence in antidiabetic drug discovery: The advances in QSAR and the prediction of α-glucosidase inhibitors Computational and Structural Biotechnology Journal 23 2964 2977 10.1016/j.csbj.2024.07.003 39148608 PMC11326494

[b164-tjb-49-05-498] Ou L Setegne MT Elliot J Shen F Dassama LMK 2025 ) Protein-Based Degraders: From Chemical Biology Tools to Neo-Therapeutics Chemical Reviews 10.1021/acs.chemrev.4c00595 PMC11870016 39818743

[b165-tjb-49-05-498] Oviedo F Ferres JL Buonassisi T Butler KT 2022 Interpretable and Explainable Machine Learning for Materials Science and Chemistry Accounts of Materials Research 3 6 597 607 10.1021/accountsmr.1c00244

[b166-tjb-49-05-498] Pan J Liu W Zhu J Zhou J Zhang W 2025 RefineScore: Improving Ligand Docking Accuracy and Interpretability by Predicting MDN Corrective Physical Interactions 10.26434/chemrxiv-2025-kbhfb-v2

[b167-tjb-49-05-498] Pandey VK Tripathi A Taufeeq A Dar AH Samrot AV 2024 Significance and applications of carbon dots in anti cancerous nanodrug conjugate development: A review Applied Surface Science Advances 19 100550 10.1016/j.apsadv.2023.100550

[b168-tjb-49-05-498] Parashar AK Saraogi GK Jain PK Kurmi B Shrivastava V 2024 Polymer-drug conjugates: revolutionizing nanotheranostic agents for diagnosis and therapy Discover Oncology 15 1 1 21 10.1007/S12672-024-01509-9 39527173 PMC11554983

[b169-tjb-49-05-498] Park J Kim YM Hong S Han B Nam KT 2023 Closed-loop optimization of nanoparticle synthesis enabled by robotics and machine learning Matter 6 3 677 690 10.1016/j.matt.2023.01.018

[b170-tjb-49-05-498] Parvin N Joo SW Mandal TK 2025 Nanomaterial-Based Strategies to Combat Antibiotic Resistance: Mechanisms and Applications Antibiotics 14 2 10.3390/antibiotics14020207 PMC11852044 40001450

[b171-tjb-49-05-498] Patra JK Das G Fraceto LF Campos EVR Rodriguez-Torres MDP 2018 Nano based drug delivery systems: recent developments and future prospects Journal of Nanobiotechnology 16 1 71 10.1186/S12951-018-0392-8 30231877 PMC6145203

[b172-tjb-49-05-498] Patyal R Warjurkar K Sharma V 2025 Advancements in Tumor Diagnostics through Carbon Dot- Assisted Photoacoustic Imaging Advanced Optical Materials 13 4 2402343 10.1002/adom.202402343

[b173-tjb-49-05-498] Prasanna Babu Racheeti YR 2024 Review on Green Synthesis of Nanomaterials: Sustainable Approaches and Multifaceted Applications International Journal of Pharmaceutical Sciences 2 10.5281/ZENODO.10853399

[b174-tjb-49-05-498] Puech P Kandara M Paredes G Moulin L Weiss-Hortala E 2019 Analyzing the Raman Spectra of Graphenic Carbon Materials from Kerogens to Nanotubes: What Type of Information Can Be Extracted from Defect Bands? C 5 4 69 10.3390/C5040069

[b175-tjb-49-05-498] Qureshi ZA Dabash H Ponnamma D Abbas MKG 2024 Carbon dots as versatile nanomaterials in sensing and imaging: Efficiency and beyond Heliyon 10 11 e31634 10.1016/J.HELIYON.2024.E31634 38832274 PMC11145243

[b176-tjb-49-05-498] Ram TB Krishnan S Jeevanandam J Danquah MK Thomas S 2024 Emerging Biohybrids of Aptamer-Based Nano-Biosensing Technologies for Effective Early Cancer Detection Molecular Diagnosis and Therapy 28 4 425 453 10.1007/s40291-024-00717-x 38775897

[b177-tjb-49-05-498] Rana A Adhikary M Singh PK Das BC Bhatnagar S 2023 “Smart” drug delivery: A window to future of translational medicine Frontiers in Chemistry 10 1095598 36688039 10.3389/fchem.2022.1095598 PMC9846181

[b178-tjb-49-05-498] Rao A Grzelczak M 2024 Revisiting El-Sayed Synthesis: Bayesian Optimization for Revealing New Insights during the Growth of Gold Nanorods Chemistry of Materials 36 5 2577 2587 10.1021/acs.chemmater.4c00271 38680830 PMC11049742

[b179-tjb-49-05-498] Ratre P Nazeer N Soni N Kaur P Tiwari R 2024 Smart carbon-based sensors for the detection of non-coding RNAs associated with exposure to micro(nano)plastics: an artificial intelligence perspective Environmental Science and Pollution Research 31 6 8429 8452 10.1007/s11356-023-31779-9 38182954

[b180-tjb-49-05-498] Ren J Opoku H Tang S Edman L Wang J 2024 ) Carbon Dots: A Review with Focus on Sustainability Advanced Science 10.1002/advs.202405472 PMC11425242 39023174

[b181-tjb-49-05-498] Revelou PK Tsakali E Batrinou A Strati IF 2025 Applications of Machine Learning in Food Safety and HACCP Monitoring of Animal-Source Foods Foods 14 6 10.3390/foods14060922 PMC11941095 40231903

[b182-tjb-49-05-498] Rocco D Moldoveanu VG Feroci M Bortolami M Vetica F 2023 Electrochemical Synthesis of Carbon Quantum Dots ChemElectroChem 10 3 e202201104 10.1002/CELC.202201104 37502311 PMC10369859

[b183-tjb-49-05-498] Rojas K Verdugo-Molinares MG Vallejo-Cardona AA 2024 Use of encapsulating polymers of active compounds in the pharmaceutical and food industry Food Chemistry Advances 4 100619 10.1016/J.FOCHA.2024.100619

[b184-tjb-49-05-498] Roncaglia C Ferrando R 2023 Machine Learning Assisted Clustering of Nanoparticle Structures Journal of Chemical Information and Modeling 63 2 459 473 10.1021/acs.jcim.2c01203 36597194 PMC9875306

[b185-tjb-49-05-498] Roper C Tanguay RL 2018 Zebrafish as a Model for Developmental Biology and Toxicology Handbook of Developmental Neurotoxicology 143 151 10.1016/B978-0-12-809405-1.00012-2

[b186-tjb-49-05-498] Roy A Samanta S Singha K Maity P Kumari N 2020 Development of a Thermoresponsive Polymeric Composite Film Using Cross-Linked β-Cyclodextrin Embedded with Carbon Quantum Dots as a Transdermal Drug Carrier ACS Applied Bio Materials 3 5 3285 3293 doi: 10.1021/acsabm.0c00246 35025371

[b187-tjb-49-05-498] Rumondor ACF Dhareshwar SS Kesisoglou F 2016 Amorphous Solid Dispersions or Prodrugs: Complementary Strategies to Increase Drug Absorption Journal of Pharmaceutical Sciences 105 9 2498 2508 10.1016/j.xphs.2015.11.004 26886316

[b188-tjb-49-05-498] Saarela M Podgorelec V 2024 Recent Applications of Explainable AI (XAI): A Systematic Literature Review Applied Sciences 14 19 8884 10.3390/app14198884

[b189-tjb-49-05-498] Sahana S Gautam A Singh R Chandel S 2023 A recent update on development, synthesis methods, properties and application of natural products derived carbon dots Natural Products and Bioprospecting 13 1 1 21 10.1007/S13659-023-00415-X 37953431 PMC10641086

[b190-tjb-49-05-498] Sakdaronnarong C Sangjan A Boonsith S Kim DC Shin HS 2020 Recent Developments in Synthesis and Photocatalytic Applications of Carbon Dots Catalysts 10 3 320 10.3390/CATAL10030320

[b191-tjb-49-05-498] Saputra AMA Piliang AFR Dellyansyah Marpongahtun Andriayani 2024 Synthesis, properties, and utilization of carbon quantum dots as photocatalysts on degradation of organic dyes: A mini review Catalysis Communications 187 106914 10.1016/J.CATCOM.2024.106914

[b192-tjb-49-05-498] Sarma DD Santra PK Mukherjee S Nag A 2013 X-ray photoelectron spectroscopy: A unique tool to determine the internal heterostructure of nanoparticles Chemistry of Materials 25 8 1222 1232 10.1021/CM303567D

[b193-tjb-49-05-498] Sarmanova O Laptinskiy K Burikov S Dolenko S Trushina D 2022 Decoding Optical Spectra with Neural Networks to Monitor the Elimination of Carbon Nanoagents from the Body Optical Memory and Neural Networks (Information Optics) 31 3 256 265 10.3103/S1060992X22030109

[b194-tjb-49-05-498] Senanayake RD Yao X Froehlich CE Cahill MS Sheldon TR 2022 Machine Learning-Assisted Carbon Dot Synthesis: Prediction of Emission Color and Wavelength Journal of Chemical Information and Modeling 62 23 5918 5928 10.1021/acs.jcim.2c01007 36394850 PMC9749762

[b195-tjb-49-05-498] Sengar P Chauhan K Hirata GA 2022 Progress on carbon dots and hydroxyapatite based biocompatible luminescent nanomaterials for cancer theranostics Translational Oncology 24 101482 10.1016/j.tranon.2022.101482 35841822 PMC9293661

[b196-tjb-49-05-498] Serrano BA Gheorghe LC Exner TE Resch S Wolf C 2024 The role of FAIR nanosafety data and nanoinformatics in achieving the UN sustainable development goals: the NanoCommons experience RSC Sustainability 2 5 1378 1399 10.1039/d3su00148b

[b197-tjb-49-05-498] Sezer E Dokuzparmak E Özçelik H Yaşar E Kaya T 2025 Harnessing Machine Learning to Revolutionize Electrochemical Detection of Vitamin E Acetate in E-Liquids ACS Omega 10.1021/acsomega.5c02363 PMC12223865 40621006

[b198-tjb-49-05-498] Shabbir H Csapó E Wojnicki M 2023 Carbon Quantum Dots: The Role of Surface Functional Groups and Proposed Mechanisms for Metal Ion Sensing Inorganics 11 6 262 10.3390/INORGANICS11060262

[b199-tjb-49-05-498] Shang J Zhou Q Wang K Wei Y 2024 Engineering of Green Carbon Dots for Biomedical and Biotechnological Applications Molecules 29 18 4508 10.3390/MOLECULES29184508 39339503 PMC11434350

[b200-tjb-49-05-498] Sheikh M Jirvankar PS 2024 Harnessing artificial intelligence for enhanced nanoparticle design in precision oncology AIMS Bioengineering 11 4 574 597 10.3934/bioeng.2024026

[b201-tjb-49-05-498] Shirokii N Din Y Petrov I Seregin Y Sirotenko S 2023 Quantitative Prediction of Inorganic Nanomaterial Cellular Toxicity via Machine Learning Small 19 19 10.1002/smll.202207106 36772908

[b202-tjb-49-05-498] Singh AV Varma M Laux P Choudhary S Datusalia AK 2023 Artificial intelligence and machine learning disciplines with the potential to improve the nanotoxicology and nanomedicine fields: a comprehensive review Archives of Toxicology 97 4 963 979 10.1007/s00204-023-03471-x 36878992 PMC10025217

[b203-tjb-49-05-498] Singh B Adcock AF Dumra S Collins J Yang L 2025 Microwave-Assisted Carbonization Processing for Carbon Dot-like Nanomaterials with Antimicrobial Properties Micro 5 1 14 10.3390/MICRO5010014

[b204-tjb-49-05-498] Singh I Arora R Dhiman H Pahwa R 2018 Carbon Quantum Dots: Synthesis, Characterization and Biomedical Applications Turkish Journal of Pharmaceutical Sciences 15 2 219 230 10.4274/TJPS.63497 32454664 PMC7228020

[b205-tjb-49-05-498] Skrzydlewski P Twarużek M Grajewski J 2022 Cytotoxicity of Mycotoxins and Their Combinations on Different Cell Lines: A Review Toxins 14 4 244 35448853 10.3390/toxins14040244 PMC9031280

[b206-tjb-49-05-498] Speranza G 2022 Characterization of Carbon Nanostructures by Photoelectron Spectroscopies Materials 15 13 4434 10.3390/MA15134434 35806559 PMC9267296

[b207-tjb-49-05-498] Suk JS Xu Q Kim N Hanes J Ensign LM 2015 PEGylation as a strategy for improving nanoparticle-based drug and gene delivery Advanced Drug Delivery Reviews 99 Pt A 28 10.1016/J.ADDR.2015.09.012 26456916 PMC4798869

[b208-tjb-49-05-498] Sun W Hu Q Ji W Wright G Gu Z 2017 Leveraging physiology for precision drug delivery Physiological Reviews 97 1 189 225 10.1152/PHYSREV.00015.2016

[b209-tjb-49-05-498] Sun Y Zheng S Liu L Kong Y Zhang A 2020 The Cost-Effective Preparation of Green Fluorescent Carbon Dots for Bioimaging and Enhanced Intracellular Drug Delivery Nanoscale Research Letters 15 1 1 9 10.1186/s11671-020-3288-0 32130552 PMC7056761

[b210-tjb-49-05-498] Surana K Bhattacharya B 2021 Fluorescence Quenching by Förster Resonance Energy Transfer in Carbon-Cadmium Sulfide Core-Shell Quantum Dots ACS Omega 6 48 32749 32753 doi: 10.1021/acsomega.1c04529 34901623 PMC8655935

[b211-tjb-49-05-498] Tafreshi FA Fatahi Z Ghasemi SF Taherian A Esfandiari N 2020 Ultrasensitive fluorescent detection of pesticides in real sample by using green carbon dots PLOS ONE 15 3 e0230646 10.1371/JOURNAL.PONE.0230646 32208468 PMC7092965

[b212-tjb-49-05-498] Tavernaro I Matiushkina A Rother KS Mating C Resch-Genger U 2024 ) Exploring the potential of simple automation concepts for quantifying functional groups on nanomaterials with optical assays Nano Research 10.1007/s12274-024-6970-1

[b213-tjb-49-05-498] Teame T Zhang Z Ran C Zhang H Yang Y 2019 The use of zebrafish (Danio rerio) as biomedical models Animal Frontiers: The Review Magazine of Animal Agriculture 9 3 68 10.1093/AF/VFZ020 32002264 PMC6951987

[b214-tjb-49-05-498] Tegafaw T Mulugeta E Zhao D Liu Y Chen X 2025 Surface Modification, Toxicity, and Applications of Carbon Dots to Cancer Theranosis: A Review Nanomaterials 15 11 781 10.3390/NANO15110781 40497830 PMC12157935

[b215-tjb-49-05-498] Tejwan N Kundu M Ghosh N Chatterjee S Sharma A 2022 Synthesis of green carbon dots as bioimaging agent and drug delivery system for enhanced antioxidant and antibacterial efficacy Inorganic Chemistry Communications 139 109317 10.1016/J.INOCHE.2022.109317

[b216-tjb-49-05-498] Tian J An M Zhao X Wang Y Hasan M 2023 Advances in Fluorescent Sensing Carbon Dots: An Account of Food Analysis ACS Omega 8 10 9031 10.1021/ACSOMEGA.2C07986 36936334 PMC10018703

[b217-tjb-49-05-498] Tolosa L Donato MT Gómez-Lechón MJ 2015 General Cytotoxicity Assessment by Means of the MTT Assay Methods in Molecular Biology 1250 333 348 10.1007/978-1-4939-2074-7_26 26272156

[b218-tjb-49-05-498] Tong X Lin X Duan N Wang Z Wu S 2022 Laser-Printed Paper-Based Microfluidic Chip Based on a Multicolor Fluorescence Carbon Dot Biosensor for Visual Determination of Multiantibiotics in Aquatic Products ACS Sensors 7 12 3947 3955 10.1021/acssensors.2c02008 36454704

[b219-tjb-49-05-498] Tripathy D Mishra SK Sahu S Katiyar A Kumar N 2025 Carbon dots as smart biosensing and imaging materials delving with antimicrobial, antifungal, and antiviral potentials against infectious pathogens Journal of the Chinese Chemical Society 10.1002/jccs.202400372

[b220-tjb-49-05-498] Tropsha A Isayev O Varnek A Schneider G Cherkasov A 2024 Integrating QSAR modelling and deep learning in drug discovery: the emergence of deep QSAR Nature Reviews Drug Discovery 23 2 141 155 10.1038/s41573-023-00832-0 38066301

[b221-tjb-49-05-498] Ullal N Muthamma K Sunil D 2022 Carbon dots from eco-friendly precursors for optical sensing application: an up-to-date review Chemical Papers 76 10 6097 6127 10.1007/S11696-022-02353-3

[b222-tjb-49-05-498] Ulucan-Karnak F Kuru Cİ 2023 Advantages of nanodrug targeting than conventional dosage system Nanotechnology for Drug Delivery and Pharmaceuticals 295 310 10.1016/B978-0-323-95325-2.00003-1

[b223-tjb-49-05-498] Ulucan-Karnak F Camci-Unal G Karacaoglu B Seydibeyoğlu MÖ 2023 Design and control of nanorobots and nanomachines in drug delivery and diagnosis A handbook of artificial intelligence in drug delivery Academic Press 371 394

[b224-tjb-49-05-498] Voronin DV Abalymov AA Svenskaya YI Lomova MV 2021 Key Points in Remote-Controlled Drug Delivery: From the Carrier Design to Clinical Trials International Journal of Molecular Sciences 22 17 9149 10.3390/IJMS22179149 34502059 PMC8430748

[b225-tjb-49-05-498] Wang B Lu S 2022 The light of carbon dots: From mechanism to applications Matter 5 1 110 149 10.1016/J.MATT.2021.10.016

[b226-tjb-49-05-498] Wang C Lu L Liu Z Lai Y Li J 2023 Green Carbon Dots Illuminate Biogenic Nanohybrids toward Soft, Piezo/Photoactive, and Physically Transient Nanogenerators ACS Sustainable Chemistry and Engineering 11 40 14646 14658 10.1021/acssuschemeng.3c00957

[b227-tjb-49-05-498] Wang H Sun C Chen X Zhang Y Colvin VL 2017a Excitation wavelength independent visible color emission of carbon dots Nanoscale 9 5 1909 10.1039/C6NR09200D PMC6265045 28094404

[b228-tjb-49-05-498] Wang J Guo Y Geng X Hu J Yan M 2021 Quantitative Structure-Activity Relationship Enables the Rational Design of Lipid Droplet-Targeting Carbon Dots for Visualizing Bisphenol A-Induced Nonalcoholic Fatty Liver Disease-like Changes ACS Applied Materials and Interfaces 13 37 44086 44095 10.1021/acsami.1c13157 34516075

[b229-tjb-49-05-498] Wang L Wang H Bai M Wu Y Guo T 2024 ) A comparative review: research in safety and sustainability of carbon nanomaterials without and with machine learning assistance IEEE Access 10.1109/ACCESS.2024.3494549 PMC11767896 39858671

[b230-tjb-49-05-498] Wang Q Zhang Q He H Feng Z Mao J 2022 Carbon Dot Blinking Fingerprint Uncovers Native Membrane Receptor Organizations via Deep Learning Analytical Chemistry 94 9 3914 3921 10.1021/acs.analchem.1c04947 35188385

[b231-tjb-49-05-498] Wang W Zhang Y Wang YB 2014 Noncovalent π×××π interaction between graphene and aromatic molecule: Structure, energy, and nature Journal of Chemical Physics 140 9 10.1063/1.4867071 24606356

[b232-tjb-49-05-498] Wang Y Cui Y Zhao Y He B Shi X 2017b Fluorescent carbon dot-gated multifunctional mesoporous silica nanocarriers for redox/enzyme dual-responsive targeted and controlled drug delivery and real-time bioimaging European Journal of Pharmaceutics and Biopharmaceutics 117 105 115 10.1016/j.ejpb.2017.03.019 28363599

[b233-tjb-49-05-498] Wang Y Wang Q Liu W Xin X Zhao B 2025 A Review on the Synthesis of Carbon Dots and Their Applications in Environmental Analysis Crystals 15 5 384 10.3390/CRYST15050384

[b234-tjb-49-05-498] Wasilewski T Kamysz W Gębicki J 2024 AI-Assisted Detection of Biomarkers by Sensors and Biosensors for Early Diagnosis and Monitoring Biosensors 14 7 356 10.3390/BIOS14070356 39056632 PMC11274923

[b235-tjb-49-05-498] Wierling A Schwanitz VJ Altinci S Bałazińska M Barber MJ 2021 Fair metadata standards for low carbon energy research—a review of practices and how to advance Energies 14 20 10.3390/en14206692

[b236-tjb-49-05-498] Wu H Su W Xu H Zhang Y Li Y 2021 Applications of carbon dots on tumour theranostics VIEW, 2 2 10.1002/VIW.20200061

[b237-tjb-49-05-498] Wu L Cai X Nelson K Xing W Xia J 2013 A Green Synthesis of Carbon Nanoparticle from Honey for Real-Time Photoacoustic Imaging Nano Research 6 5 312 10.1007/S12274-013-0308-8 23824757 PMC3696503

[b238-tjb-49-05-498] Xie C Qiu H Liu L You Y Li H 2025 Machine Learning Approaches in Polymer Science: Progress and Fundamental for a New Paradigm SmartMat 6 1 10.1002/smm2.1320

[b239-tjb-49-05-498] Xin AW Rivera-Delgado E von Recum HA 2024 Using QSAR to predict polymer-drug interactions for drug delivery Frontiers in Soft Matter 4 10.3389/frsfm.2024.1402702

[b240-tjb-49-05-498] Xing C Chen G Zhu X An J Bao J 2024 Synthesis of carbon dots with predictable photoluminescence by the aid of machine learning Nano Research 17 3 1984 1989 10.1007/s12274-023-5893-6

[b241-tjb-49-05-498] Xu J Huang BB Lai CM Lu YS Shao JW 2024 Advancements in the synthesis of carbon dots and their application in biomedicine Journal of Photochemistry and Photobiology B: Biology 255 112920 10.1016/J.JPHOTOBIOL.2024.112920 38669742

[b242-tjb-49-05-498] Xu X Ray R Gu Y Ploehn HJ Gearheart L Raker K Scrivens WA 2004 Electrophoretic analysis and purification of fluorescent single-walled carbon nanotube fragments Journal of the American Chemical Society 126 40 12736 12737 10.1021/ja040082h 15469243

[b243-tjb-49-05-498] Xuan L Ju Z Skonieczna M Zhou PK Huang R 2023 Nanoparticles-induced potential toxicity on human health: Applications, toxicity mechanisms, and evaluation models MedComm 4 4 e327 10.1002/MCO2.327 37457660 PMC10349198

[b244-tjb-49-05-498] Yadav N Pandey S Gupta A Dudani P Gupta S Rangarajan K 2023 Data Privacy in Healthcare: In the Era of Artificial Intelligence Indian dermatology online journal, 14 6 788 792 10.4103/idoj.idoj_543_23 38099022 PMC10718098

[b245-tjb-49-05-498] Yan X Yue T Winkler DA Yin Y Zhu H 2023 Converting Nanotoxicity Data to Information Using Artificial Intelligence and Simulation Chemical Reviews 123 13 8575 8637 10.1021/acs.chemrev.3c00070 37262026

[b246-tjb-49-05-498] Yang HL Bai LF Geng ZR Chen H Xu LT 2023 Carbon quantum dots: Preparation, optical properties, and biomedical applications Materials Today Advances, 18 100376 10.1016/J.MTADV.2023.100376

[b247-tjb-49-05-498] Ye Z Ouyang D 2024 Opportunities and Challenges of Artificial Intelligence (AI) in Drug Delivery Exploring Computational Pharmaceutics - Ai and Modeling in Pharma 4 0 10 58 10.1002/9781119987260.ch2

[b248-tjb-49-05-498] Yu R Ou M Hou Q Li C Qu S 2025 Metal and non-metal doped carbon dots: properties and applications Light: Advanced Manufacturing 5 4 647 666 10.37188/LAM.2024.041

[b249-tjb-49-05-498] Zelzer M Todd SJ Hirst AR McDonald TO Ulijn RV 2012 Enzyme responsive materials: design strategies and future developments Biomaterials Science 1 1 11 39 10.1039/C2BM00041E 32481995

[b250-tjb-49-05-498] Zeng F Fan Z Li S Li L Sun T 2023 Tumor Microenvironment Activated Photoacoustic-Fluorescence Bimodal Nanoprobe for Precise Chemo-immunotherapy and Immune Response Tracing of Glioblastoma ACS Nano 17 20 19753 19766 10.1021/acsnano.3c03378 37812513

[b251-tjb-49-05-498] Zhang D Wada H 2021 Laser Ablation in Liquids for Nanomaterial Synthesis and Applications Handbook of Laser Micro-and Nano-Engineering 3 1481 1515 10.1007/978-3-030-63647-0_30

[b252-tjb-49-05-498] Zhang Q Tao Y Tang B Yang J Liang H 2022 Graphene quantum dots with improved fluorescence activity via machine learning: Implications for fluorescence monitoring ACS Applied Nano Materials 5 2 2728 2737

[b253-tjb-49-05-498] Zhang X Chen Z Chen F Fanady B Wang B 2025 Material intelligence by the convergence of artificial intelligence and robotic platforms Nexus 2 3 100083

[b254-tjb-49-05-498] Zhao Q Wang S Yang Y Li X Di D 2017 Hyaluronic acid and carbon dots-gated hollow mesoporous silica for redox and enzyme-triggered targeted drug delivery and bioimaging Materials Science and Engineering C 78 475 484 10.1016/j.msec.2017.04.059 28576012

[b255-tjb-49-05-498] Zhong Y Chen L Yu S Yang Y Liu X 2023 Advances in Magnetic Carbon Dots: A Theranostics Platform for Fluorescence/Magnetic Resonance Bimodal Imaging and Therapy for Tumors ACS Biomaterials Science and Engineering 9 12 6548 6566 10.1021/acsbiomaterials.3c00988 37945516

[b256-tjb-49-05-498] Zhou Y Wang Y Peijnenburg W Vijver MG Balraadjsing S 2024 Application of Machine Learning in Nanotoxicology: A Critical Review and Perspective Environmental Science and Technology 10.1021/acs.est.4c03328 39109992

[b257-tjb-49-05-498] Zhuo Y Zhao YG Zhang Y 2024 Enhancing Drug Solubility, Bioavailability, and Targeted Therapeutic Applications through Magnetic Nanoparticles Molecules 29 20 4854 10.3390/MOLECULES29204854 39459222 PMC11510236

[b258-tjb-49-05-498] Zuhair V Babar A Ali R Oduoye MO Noor Z 2024 Exploring the Impact of Artificial Intelligence on Global Health and Enhancing Healthcare in Developing Nations Journal of Primary Care & Community Health 15 21501319241245850 10.1177/21501319241245847 PMC11010755 38605668

